# National Acceptance and Determinants of Immersive Extended Reality in Health Care in China: Cross-Sectional Study

**DOI:** 10.2196/88000

**Published:** 2026-06-26

**Authors:** Jiaying Li, Patricia M Davidson, Helen YL Chan, Cho Lee Wong, Xiang Qi, Zengjie Ye, Ankie Tan Cheung, Daniel YT Fong, Yibo Wu, Junxin Li

**Affiliations:** 1 The Nethersole School of Nursing, Faculty of Medicine, The Chinese University of Hong Kong, Hong Kong, China (Hong Kong); 2 University of New South Wales Sydney, Sydney, Australia; 3 Rory Meyers College of Nursing, New York University, New York, NY, United States; 4 School of Nursing, Guangzhou Medical University, Guangzhou, China; 5 School of Nursing, Li Ka Shing Faculty of Medicine, The University of Hong Kong, 5/F, HKUMed Academic Building, 3 Sassoon Road, Pokfulam, Hong Kong, 999077, China (Hong Kong), 852 39176645; 6 Department of Nursing, The Fourth Affiliated Hospital of School of Medicine, International School of Medicine, International Institutes of Medicine, Zhejiang University, Yiwu, China; 7 School of Nursing, Johns Hopkins University, Baltimore, MD, United States

**Keywords:** immersive health care technologies, extended reality, technology acceptance, determinants, digital health

## Abstract

**Background:**

Immersive extended reality (XR) promises to transform health care, but public acceptance and user-side determinants of acceptance remain largely unknown.

**Objective:**

This study aimed to estimate national immersive XR acceptance and identify sociodemographic, psychosocial, health, and digital determinants among adults in China.

**Methods:**

This nationwide cross-sectional survey was conducted from June to September 2024 among 35,861 Chinese adults aged 18 years or older across 33 provincial-level regions and 800 communities, using multistage sampling. Immersive XR acceptance (0‐100) and 139 potential predictors across demographic, adversity, personality, literacy, lifestyle, physical, and psychosocial domains were assessed. Poststratification weights were calibrated to the national age-sex distribution. Determinants were identified using survey-weighted hierarchical linear regression with Benjamini-Hochberg false discovery rate correction. Elastic net validation assessed predictor robustness, and classification and regression tree analysis identified profiles of likely nonacceptors.

**Results:**

Mean acceptance was 63.11 (95% CI 62.75-63.46), varying by province (95% CI 47.9‐72.3). Acceptance was highest in younger adults (women aged 18‐24 years and men aged 30‐34 years) and declined with age; sex differences were minimal. Acceptance varied across 15 chronic conditions (lowest: rare diseases, mean 54.24, 95% CI 45.40‐63.07; highest: hyperlipidemia, mean 62.26, 95% CI 60.19-64.33). Acceptance was strongly associated with socioeconomic factors (higher social status: standardized β=0.17, 95% CI 0.16-0.18; higher youth socioeconomic status: standardized β=0.14, 95% CI 0.13-0.16; better youth economic environment: standardized β=0.06, 95% CI 0.04-0.07), digital capital (prior digital health use: standardized β=0.15, 95% CI 0.14-0.16; eHealth literacy: standardized β=0.09, 95% CI 0.07-0.10), and key traits (self-efficacy: standardized β=0.10, 95% CI 0.09-0.12; personal meaning: standardized β=0.06, 95% CI 0.04-0.07). Other positive predictors included having 2 types of medical insurance (standardized β=0.07, 95% CI 0.04-0.09), stable sleep duration (standardized β=0.07, 95% CI 0.03-0.10), and childhood psychological abuse (standardized β=0.07, 95% CI 0.05‐0.09). Strong negative predictors included older age (standardized β=−0.08, 95% CI −0.10 to −0.06), couple-only household (standardized β=−0.08, 95% CI −0.11 to −0.04), childhood sexual abuse (standardized β=−0.08, 95% CI −0.10 to −0.06), attention-deficit/hyperactivity disorder (standardized β=−0.07, 95% CI −0.09 to −0.05), more siblings (standardized β=−0.07, 95% CI −0.08 to −0.05), childhood physical abuse (standardized β=−0.05, 95% CI −0.06 to −0.03), and collective violence exposure (standardized β=−0.05, 95% CI −0.07 to −0.03). The 6-node classification tree showed modest discrimination (test area under the curve=0.61; accuracy=0.681), high specificity (specificity=0.903), and low sensitivity (sensitivity=0.242), suggesting better identification of likely nonacceptors than likely acceptors.

**Conclusions:**

Acceptance of immersive XR in health care in China was moderate but uneven. Adoption varied by age, region, socioeconomic resources, digital capital, psychosocial factors, household context, health status, and adversity exposure, suggesting that XR implementation is both a digital health innovation and a health equity challenge. Deployment should include targeted education, accessible demonstrations, usability support, and trusted guidance for less accepting groups, especially older adults, socioeconomically disadvantaged groups, people with limited digital health experience, and psychosocially vulnerable populations.

## Introduction

Immersive extended reality (XR) technologies—encompassing virtual, augmented, and mixed reality (VR, AR, and MR)—are increasingly used in health care for rehabilitation, symptom management, procedural support, and training [1-3]. Recent evidence syntheses show that XR is now being tested across diverse clinical areas, including pain, neurorehabilitation, cancer-supportive care, mental health, older adult care, and health care education [4-8]. Although benefits have been reported for outcomes such as pain, anxiety, fatigue, and functional impairment, evidence quality and generalizability remain uneven [4-8]. With a market projected to exceed US $5 billion by 2030 (>25% annual growth) [1], and as clinical applications expand, the key implementation question is no longer solely whether immersive XR is clinically effective but also whether it is acceptable to intended users in real-world health care contexts. This issue is particularly important because immersive XR is not a low-friction digital tool. It often requires head-mounted devices, embodied interaction, sensory immersion, user confidence, and clinical or home-based support [9-11]. As a result, even clinically promising XR interventions may fail to scale if users are unwilling to adopt them.

Population-level evidence is needed to guide the real-world scale-up of immersive XR in health care. However, current evidence on XR acceptance remains fragmented and is drawn largely from small or highly specific cohorts, leaving broader population patterns unclear [12-16]. Existing evidence highlights stark contrasts between lay and professional perspectives, as well as across differing cultural and clinical contexts [12-16]. For instance, a gap exists even in high-income settings: general public awareness remains low and only 18.3% of UK adults recognize VR’s clinical applications [17], while professional clinical integration also lags, with only 7.1% of 658 US rehabilitation specialists routinely using VR by 2025 [13]. Among patients, receptivity demonstrates similarly deep divisions: while 90% of patients in the United States who experience chronic pain express willingness to join a VR trial [14], Chinese oncology patients remain ambivalent [15], yet obese Chinese individuals show strong intent to try VR-based exergaming [16]. Although these studies provide useful evidence on XR acceptance in specific groups, their findings may not reflect broader public attitudes. In particular, they do not clarify how acceptance varies across geographic regions, age-sex groups, or people living with different noncommunicable diseases. This limits the ability of policymakers, clinicians, and technology developers to identify which populations are most likely to accept or resist immersive XR in health care.

Beyond estimating overall acceptance, understanding the determinants of immersive XR acceptance is essential for designing accessible interventions and guiding equitable implementation. However, current evidence remains fragmented [9,18-24]. Existing evidence suggests that acceptance may be lower among older adults [18,19], higher among women [20], and lower in groups with less education or minority status [21,22]. Psychological factors also appear relevant: openness and technology self-efficacy have been associated with greater acceptance [9,23], whereas technology anxiety may deter use [24]. Yet these studies usually examine a narrow set of determinants and rarely assess broader upstream factors that may shape digital health acceptance before users encounter a specific XR system. While established frameworks such as the Technology Acceptance Model and the Unified Theory of Acceptance and Use of Technology effectively capture immediate, system-specific interactions [25], they are less suited to capturing wider biopsychosocial and equity-related characteristics, such as social resources, prior technology exposure, health literacy, perceived health needs, lifestyle, psychological vulnerability, and adverse life experiences. A broader biopsychosocial lens is therefore needed to understand not only whether the public is willing to use immersive XR in health care but also which population groups may be left behind during implementation.

To address the limitations of prior small, condition-specific, and nonnational studies, we conducted what is, to our knowledge, the largest national survey of health care XR acceptance to date. Our study is distinguished by its national scope; assessment of 139 determinants across socioeconomic, psychosocial, experiential, and health-related domains; equity-oriented analysis; and development of a practical tool to identify likely nonacceptors. Specifically, our main aims were to (1) estimate national levels of immersive XR acceptance and map geographic, age-sex, and noncommunicable disease-specific patterns; (2) evaluate 139 candidate determinants spanning socioeconomics, childhood adversity, lifestyle, health literacy, and psychosocial factors; and (3) develop and validate a streamlined classification tree to flag profiles of immersive XR nonacceptors.

We hypothesized that immersive XR acceptance varies significantly by geography, age, and disease cohort, and those biopsychosocial factors independently drive nonacceptance, converging into distinct, classifiable user profiles.

## Methods

### Study Design

This study was a nationwide, population-based, and cross-sectional survey conducted in China between June and September 2024 as part of the Psychology and Behavior Investigation of Chinese Residents program.

### Study Setting

The study was conducted across 33 provincial regions in China as part of a national psychological and behavioral health program. Data collection took place between June 23 and September 29, 2024, at the selected survey sites.

### Participants

#### Inclusion and Exclusion

Eligible participants were Chinese nationals who were aged 18 years or older, resided in China for at least 11 months per year, were able to complete the online questionnaire, and provided informed consent. We excluded individuals with psychosis, cognitive impairment, or those with prior participation in similar surveys.

#### Participant Characteristics

The analytic sample had a mean age of 47.50 (SD 16.74) years; 43.0% (15,428/35,861) were male, 56.5% (20,275/35,861) were female, and 0.4% (158/35,861) identified as other sex.

### Sampling Procedures

To improve national coverage of Chinese adults, we used a 5-stage geographic sampling framework across 33 provincial-level administrative regions. The sampling process combined multistage area selection with quota-based recruitment at the final stage. Specifically, 150 cities, 202 districts or counties, 390 townships or streets, and 800 communities or villages were selected through multiple geographic stages, after which participant recruitment within selected communities or villages was guided by demographic quota control. Poststratification raking weights were then applied to align the sample with the national age- and sex-specific distribution.

### Sample Size, Power, and Precision

Of 38,793 questionnaires distributed, 38,424 (99.0%) were returned; after exclusions, 36,240 valid cases remained. A final 2-investigator audit yielded 35,861 records for analysis. Given this large national sample, the study was expected to provide stable estimates and reasonably narrow CIs for the primary descriptive and regression analyses. Only 4 of 139 predictors had missing data (range 1.54%‐15.30%); the outcome and all other predictors were complete. We therefore used complete-case analysis and did not perform multiple imputation.

### Measures and Covariates

#### Primary Outcome

The primary outcome was self-rated acceptance of immersive health care technologies, measured on a 0 (completely unacceptable) to 100 (completely acceptable) scale. Acceptance was defined as participants’ willingness to use immersive XR (VR, AR, or MR) in health care. Participants were provided with standardized definitions of each modality to ensure comprehension.

#### Exposures, Predictors, and Covariates

We assessed 139 candidate variables as potential exposures, predictors, and covariates across 7 prespecified domains. To improve readability, the main text summarizes each domain only; full instrument names, acronyms, scoring ranges, item details, and reliability metrics are provided in Table S1 in Multimedia Appendix 1.

Specifically, block 1 (demographics and socioeconomics) included 26 variables: age, sex, gender identity, sexual orientation, handedness, race, religion, education, occupation, income, city tier, Hukou registration, perceived social status, relationship status, family structure, living alone, siblings, dwelling area, number of rooms, assets, debt, urban or rural status, years in community, insurance coverage, insurance enrollment site, and difficulty paying medical bills. Block 2 (life adversity and stress) included 30 variables covering 14 past year stressors, 15 adverse childhood experiences, and perceived childhood socioeconomic environment. Block 3 (personality and self-efficacy) included 7 variables: extraversion, agreeableness, conscientiousness, neuroticism, openness, general self-efficacy, and narcissistic admiration or rivalry. Block 4 (health literacy and empowerment) included 15 variables: family-neighbor relationships, presence of a confidant, receipt of tangible help, perceived social support, social isolation, loneliness, social connection, family health, family communication, eHealth literacy, general health literacy, antibiotic knowledge, implicit health beliefs, eating self-regulation, and prior digital health use. Block 5 (lifestyle behaviors) included 14 variables: tobacco use, alcohol consumption, physical activity, chronotype, sleep duration, sleep difficulty, snoring, daytime sleepiness, sleep stability, salt-adding habits, and engagement in music, visual arts, dance, and other arts. Block 6 (physical health and exposures) included 37 variables: BMI; 14 physician-diagnosed chronic diseases; 14 types of injuries in the past 14 months; vaccination status for human papillomavirus, influenza, shingles, hepatitis B virus, and COVID-19; number of COVID-19 infections; no vaccinations; and overall health status. Block 7 (mental and psychosocial health) included 10 variables: depressive symptoms, anxiety, perceived stress, attention-deficit/hyperactivity disorder traits, work burnout, cyberchondria, resilience, maladaptive daydreaming, social media addiction, and meaning in life.

### Data Collection and Quality of Measurements

Between June 23 and September 29, 2024, trained surveyors visited selected sites, confirmed eligibility, obtained e-consent, and administered an electronic questionnaire via Wenjuanxing. Responses were uploaded to a secure server and subjected to weekly supervisor review and automated quality control, checking for logic errors, duplicates, >20% missing data, or completion times <300 seconds.

### Data Analysis

Analyses comprised three stages: (1) descriptive summaries using poststratification raking weights, (2) multivariable modeling via 7-block hierarchical regression and elastic net validation, and (3) development of a classification and regression tree. Analyses used R (version 4.1.1; R Core Team), with 2-sided *P*<.05.

First, poststratification raking weights aligned the sample to 2020 census age-sex margins. We produced unweighted and weighted summaries (continuous: mean, SD; categorical: n [%]) and plotted weighted acceptance with 95% CIs by province, age-sex strata, single-year age (locally estimated scatterplot smoothing—smoothed by sex), and chronic condition. Province-level estimates were presented as descriptive summaries of geographic variation and were not intended to model province-level contextual effects.

Second, we fit survey-weighted hierarchical linear regression in 7 blocks—sociodemographics, life adversity and stress, personality, literacy and empowerment, lifestyle, physical health, and mental health—ordering blocks by presumed causal order to quantify independent effects while avoiding overadjustment. This hierarchical approach was used to examine whether each predictor domain explained additional variance in XR acceptance beyond domains entered earlier. For block *k*, we fitted a weighted linear model of acceptance on retained predictors from blocks 1 to *k*, applying Benjamini-Hochberg false discovery rate (FDR) correction within each block to address multiple testing, and carrying forward only predictors that remained significant after FDR correction. Continuous variables entered in original units; categorical variables were dummy-coded. Variance inflation factor >5 flagged multicollinearity. All candidate predictors were then weighted, z-standardized, and entered into a 10-fold cross-validated elastic net (α=.50) with optimal penalty λ_min_ selected by minimum mean squared error, which was used as a complementary data-driven approach because the study included a large number of potentially correlated predictors; this allowed us to assess the robustness of findings from the hierarchical regression and identify a more parsimonious set of predictors. We quantified agreement between elastic net and hierarchical selection via overlap statistics. For reporting, we prespecified the smallest effect size of interest for standardized β coefficients (β_std). Predictors with an absolute value |β_std*|* ≥0.03 were reported by name in the main text. Other FDR-significant predictors are detailed in main text tables and figures; nonsignificant predictors are tabulated in the supplement.

Third, to build a fast classification tool, we dichotomized acceptance at ≥ 80 (“acceptors”) versus <80 (“nonacceptors”) and randomly split the weighted sample 70/30 (outcome-stratified) into training and test sets. We constructed a Gini-based classification and regression tree on the training data, selecting the complexity parameter by the elbow method. For classification performance, acceptor status (score ≥80) was defined as the positive class; therefore, sensitivity represents the proportion of acceptors correctly classified and specificity represents the proportion of nonacceptors correctly classified. In the test set, we assessed discrimination using the survey-weighted receiver operating characteristic curve and its area under the curve with 1000 bootstrap CIs, and reported accuracy, sensitivity, specificity, positive predictive value, negative predictive value, and balanced accuracy at a 0.50 cutoff.

### Ethical Considerations

This study was approved by the Health-Economics and Policy Research (approval number NHC-HEPR202401) and the Shanghai Jiao Tong University Ethics Committee (approval number H20240237I). Written informed consent was obtained from all participants prior to their participation. To protect privacy and confidentiality, all study data were fully anonymized prior to analysis. Participants were not compensated for their participation. Finally, no individual participants or users can be identified in any of the images or figures included in this manuscript or its supplementary materials.

## Results

### Characteristics of Respondents

**Figure 1.**
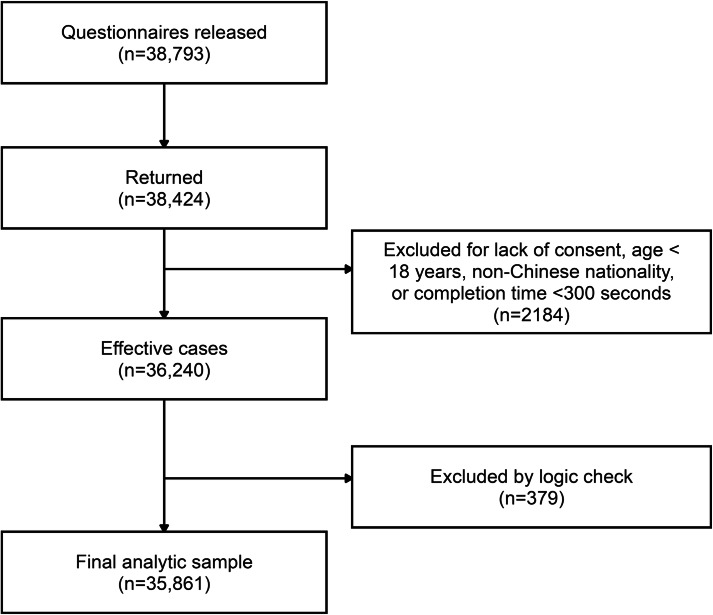
Flow diagram of data screening and participant selection in this nationwide cross-sectional survey of immersive extended reality acceptance in health care among adults in China (June-September 2024).

Among the 35,861 weighted respondents (Figure 1), mean age was 47.5 (SD 6.7) years; 50.4% (18,084/35,861) were male, 93.1% (33,381/35,861) were of Han ethnicity, 82.0% (29,409/35,861) had at least secondary education, and 72.5% (26,005/35,861) lived in urban areas. Complete sociodemographic, economic, residential, and health characteristics are shown in Table 1.

**Table 1.** Characteristics of participants included in this nationwide cross-sectional survey of immersive extended reality acceptance in health care in China, presented before and after weighting, June to September 2024 (N=35,861).

Characteristics	Unweighted (N=35,861)	Weighted (N=35,861)
Age (years), mean (SD)	34.88 (16.48)	47.50 (16.74)
Sex, n (%)		
Male	15,428 (43.0)	18,084 (50.4)
Female	20,275 (56.5)	17,639 (49.2)
Other	158 (0.4)	138 (0.4)
Race/ethnicity, n (%)		
Han ethnicity	32,228 (89.9)	33,381 (93.1)
Other	3633 (10.1)	2480 (6.9)
Religion (any), n (%)		
No	32,635 (91.0)	32,134 (89.6)
Yes	3226 (9.0)	3727 (10.4)
Education, n (%)		
Primary or below	3211 (9.0)	6452 (18.0)
Secondary	11,189 (31.2)	13,182 (36.8)
Undergraduate	19,862 (55.4)	14,416 (40.2)
Graduate	1599 (4.5)	1812 (5.1)
Employment status, n (%)		
Employed	10,449 (29.1)	13,889 (38.7)
Retired	2922 (8.1)	6677 (18.6)
Self-employed	5781 (16.1)	8615 (24.0)
Student	14,870 (41.5)	3735 (10.4)
Unemployed	1839 (5.1)	2945 (8.2)
Per capita monthly income (in Chinese yuan; CNY ¥1=US $0.148 as of June 10, 2026), n (%)		
≤1000	2068 (5.8)	1888 (5.3)
1001‐2000	3053 (8.5)	2946 (8.2)
2001‐3000	4461 (12.4)	4601 (12.8)
3001‐4000	5362 (15.0)	5547 (15.5)
4001‐5000	4789 (13.4)	4974 (13.9)
5001‐6000	4601 (12.8)	4689 (13.1)
6001‐9000	4591 (12.8)	4638 (12.9)
9001‐12,000	2945 (8.2)	2809 (7.8)
12,001‐15,000	1693 (4.7)	1610 (4.5)
≥15,001	2298 (6.4)	2160 (6.0)
City tier (by Hukou location), n (%)		
First-tier city	2052 (5.7)	2620 (7.3)
New first-tier city	4645 (13.0)	5190 (14.5)
Other city tiers	29,164 (81.3)	28,051 (78.2)
Residence in last 3 months, n (%)		
Urban	27,062 (75.5)	26,005 (72.5)
Rural	8799 (24.5)	9856 (27.5)
Household registration (Hukou), n (%)		
Urban	16,728 (46.6)	17,883 (49.9)
Rural	19,133 (53.4)	17,978 (50.1)
Medical insurance, n (%)		
None	2344 (6.5)	1661 (4.6)
Government-funded only	2779 (7.7)	2336 (6.5)
Urban resident only	14,742 (41.1)	13,021 (36.3)
Employee only	5528 (15.4)	7604 (21.2)
New rural cooperative only	3870 (10.8)	4874 (13.6)
Commercial only	547 (1.5)	475 (1.3)
2 types	4858 (13.5)	4799 (13.4)
>2 types	1193 (3.3)	1092 (3.0)
Diagnosed hypertension, n (%)		
No	33,045 (92.1)	30,174 (84.1)
Yes	2816 (7.9)	5687 (15.9)
Diagnosed diabetes, n (%)		
No	34,868 (97.2)	33,871 (94.4)
Yes	993 (2.8)	1990 (5.6)
Diagnosed hyperlipidemia, n (%)		
No	35,070 (97.8)	34,352 (95.8)
Yes	791 (2.2)	1509 (4.2)
Diagnosed coronary heart disease, n (%)		
No	35,408 (98.7)	34,923 (97.4)
Yes	453 (1.3)	939 (2.6)
Diagnosed stroke, n (%)		
No	35,751 (99.7)	35,659 (99.4)
Yes	110 (0.3)	202 (0.6)
Diagnosed respiratory disease, n (%)		
No	35,350 (98.6)	35,092 (97.9)
Yes	511 (1.4)	770 (2.1)
Diagnosed urinary disease, n (%)		
No	35,608 (99.3)	35,458 (98.9)
Yes	253 (0.7)	403 (1.1)
Diagnosed digestive disease, n (%)		
No	35,047 (97.7)	34,767 (96.9)
Yes	814 (2.3)	1094 (3.1)
Diagnosed osteoporosis, n (%)		
No	35,063 (97.8)	34,270 (95.6)
Yes	798 (2.2)	1591 (4.4)
Diagnosed arthritis, n (%)		
No	34,801 (97.0)	33,917 (94.6)
Yes	1060 (3.0)	1944 (5.4)
Diagnosed tumor, n (%)		
No	35,665 (99.5)	35,569 (99.2)
Yes	196 (0.5)	292 (0.8)
Diagnosed rare disease, n (%)		
No	35,792 (99.8)	35,790 (99.8)
Yes	69 (0.2)	71 (0.2)
Diagnosed obesity, n (%)		
No	29,153 (82.6)	27,642 (78.5)
Yes	6156 (17.4)	7585 (21.5)
Diagnosed anxiety, n (%)		
No	29,732 (82.9)	30,702 (85.6)
Yes	6129 (17.1)	5160 (14.4)
Diagnosed depressive, n (%)		
No	27,669 (77.2)	28,970 (80.8)
Yes	8192 (22.8)	6891 (19.2)
Social status score, mean (SD)	4.00 (1.35)	4.11 (1.38)

### Weighted Acceptance by Region, Age-Sex, and Chronic Condition

**Figure 2.**
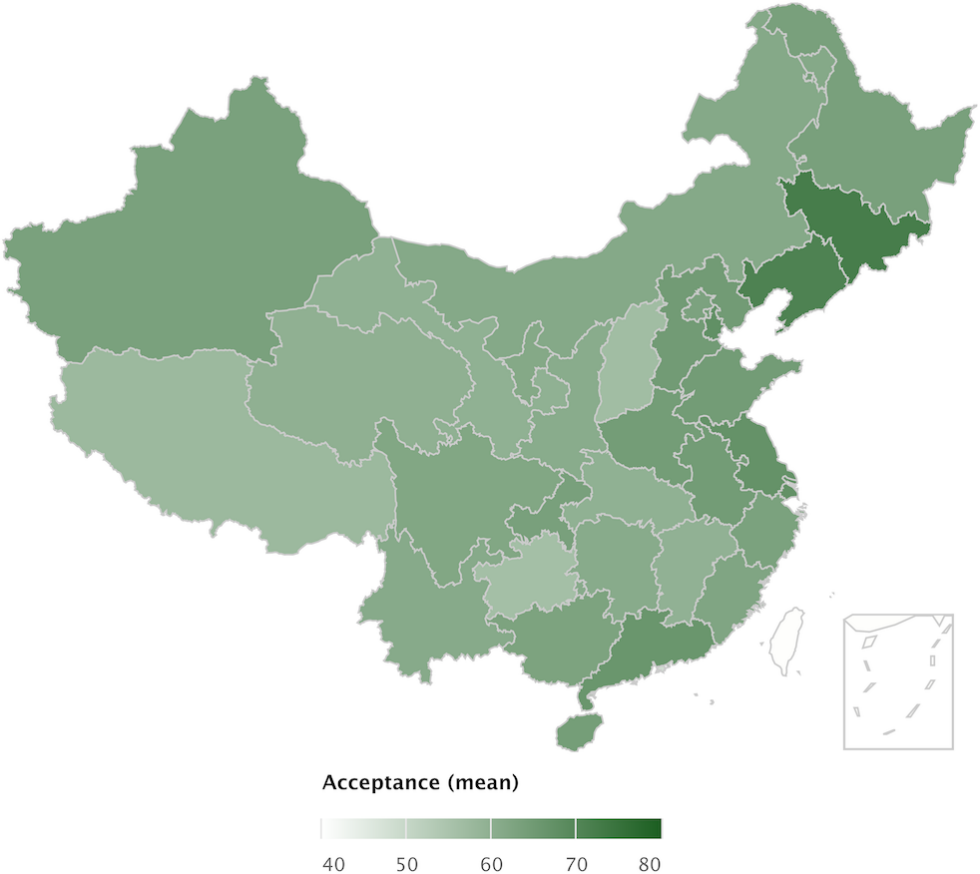
Province-level weighted mean acceptance of immersive extended reality in health care among adults in China in a nationwide cross-sectional survey conducted between June and September 2024 (N=35,861).

Overall mean weighted acceptance was 63.11 (95% CI 62.75‐63.46). Provincial means varied widely from 55.96 (95% CI 53.07-58.85) in Guizhou province to 72.34 (95% CI 66.28-78.39) in Jilin province. First-tier cities scored 63.01 (95% CI 60.92-65.10) in Beijing, 66.15 (95% CI 63.95-68.35) in Guangdong province, and 67.38 (95% CI 65.43-69.33) in Shanghai (Figure 2; Table S2 in Multimedia Appendix 1).

**Figure 3.**
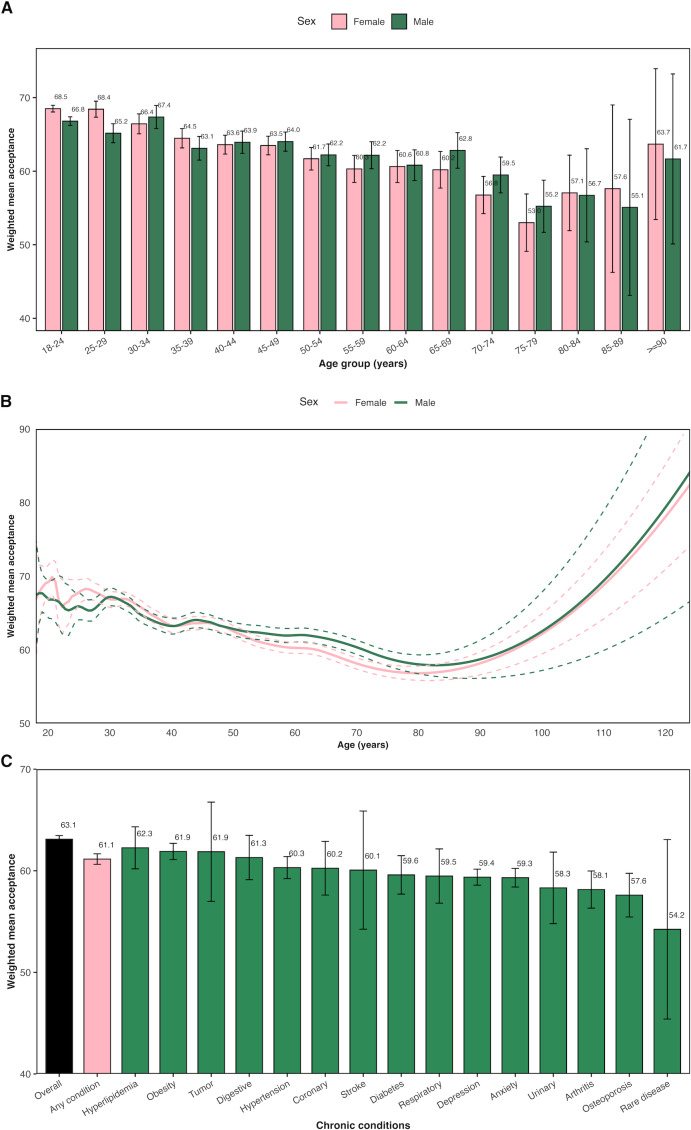
Weighted mean acceptance of immersive extended reality in health care among adults in China, shown by (A) sex and age group, (B) locally estimated scatterplot smoothing of single-year age curves stratified by sex, and (C) chronic condition, from a nationwide cross-sectional survey conducted between June and September 2024 (N=35,861). The pink bar in Figure 3C represents the mean and CI for the subgroup with at least 1 chronic condition.

Acceptance varied by age, peaking at 18‐24 years for women (mean 68.51, 95% CI 68.05‐68.96) and 30‐34 years for men (mean 66.44, 95% CI 65.09‐67.79), with a general decline thereafter. Locally estimated scatterplot smoothing curves confirmed minimal sex differences (<3 points) (Figure 3A and B; Table S3 in Multimedia Appendix 1).

Individuals with any chronic condition had lower acceptance (mean 61.15, 95% CI 60.63‐61.67) than the overall sample (mean 63.11, 95% CI 62.75‐63.46). Scores ranged from rare diseases (mean 54.24, 95% CI 45.40‐63.07) to hyperlipidemia (mean 62.26, 95% CI 60.19‐64.33). Other common conditions showed relatively similar acceptance: obesity (mean 61.90, 95% CI 61.10‐62.70), hypertension (mean 60.31, 95% CI 59.23‐61.39), diabetes (mean 59.59, 95% CI 57.70‐61.48), anxiety (mean 59.32, 95% CI 58.40‐60.23), and depression (mean 59.37, 95% CI 58.58‐60.16) (Figure 3C; Table S4 in Multimedia Appendix 1).

### Predictors of Acceptance

#### Overview

**Figure 4.**
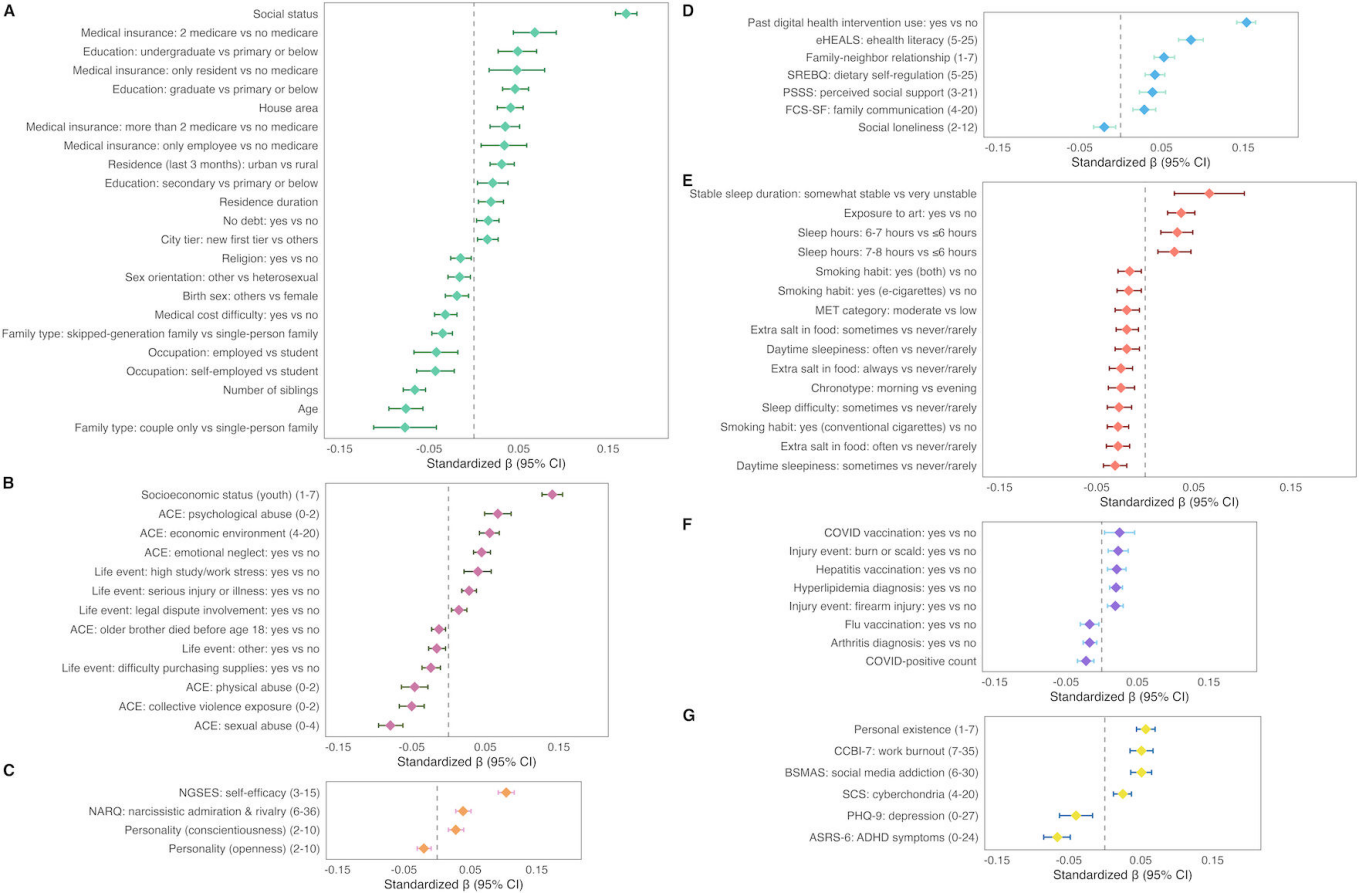
Significant predictors of immersive extended reality acceptance in health care among adults in China across 7 domains: (A) demographics and socioeconomics, (B) life adversity and stress, (C) personality, (D) health literacy and empowerment, (E) lifestyle behaviors, (F) physical health and exposures, and (G) mental health and psychosocial factors from hierarchical weighted linear regression models (N=35,861). ACE: adverse childhood experience; ADHD: attention-deficit/hyperactivity disorder; ASRS-6: 6-item Adult ADHD Self-Report Scale; BSMAS: Bergen Social Media Addiction Scale; CCBI-7: 7-item Chinese version of Copenhagen Burnout Inventory; FCS-SF: Family Communication Scale–Short Form; MET: metabolic equivalent of task; NARQ: Narcissistic Admiration and Rivalry Questionnaire; NGSES: New General Self‑Efficacy Scale; PHQ-9: 9-item Patient Health Questionnaire; PSSS: Perceived Social Support Scale; SCS: Short Cyberchondria Scale; SREBQ: Self-Regulation Eating Behavior Questionnaire.

Of 139 candidate predictors, 76 were FDR-significant. The elastic net model showed high concordance, retaining 70 of these 76 (92.1%) significant predictors. The variance inflation factor for 7 regression models ranged from 1.01 to 2.23. Fully adjusted β_std, 95% CIs, *P* values, and adjusted *P* values for FDR-significant predictors are presented in Figure 4 and Table 2. Figure 4A-G refer to significant predictors from (A) demographics and socioeconomics, (B) life adversity and stress, (C) personality, (D) health literacy and empowerment, (E) lifestyle behaviors, (F) physical health and exposures, and (G) mental health and psychosocial factors. We summarize only the strongest predictors (prespecified |β_std|≥0.03) in this section. Model statistics for nonsignificant predictors are presented in Tables S5‐S11 in Multimedia Appendix 1. Although the results are reported according to the 7 prespecified analytic blocks, these blocks were also used to inform the 4 broader domains synthesized in the *Discussion* section: blocks 1 and 2 were grouped as structural-environmental factors, blocks 3 and 7 as trait-resilience factors, block 4 as social-digital capital, and blocks 5 and 6 as behavioral-health factors.

**Table 2.** Significant predictors of immersive extended reality acceptance in health care among adults in China across 7 sequential blocks from hierarchical weighted linear regression models in a nationwide cross-sectional survey, June to September 2024 (N=35,861).

Predictors	Standardized β coefficient (95% CI)	*P* value	Adjusted *P* value
Block 1: Demographics and socioeconomics			
Social status	0.17 (0.16 to 0.18)	<.001	<.001
Medical insurance: 2 Medicare vs no Medicare	0.07 (0.04 to 0.09)	<.001	<.001
Education: graduate vs primary or below	0.05 (0.03 to 0.06)	<.001	<.001
Education: undergraduate vs primary or below^a^	0.05 (0.03 to 0.07)	<.001	<.001
Medical insurance: only resident vs no Medicare^a^	0.05 (0.02 to 0.08)	.003	.01
House area	0.04 (0.03 to 0.06)	<.001	<.001
Medical insurance: more than 2 Medicare vs no Medicare	0.04 (0.02 to 0.05)	<.001	<.001
Medical insurance: only employee vs no Medicare	0.03 (0.01 to 0.06)	.01	.03
Residence (last 3 months): urban vs rural	0.03 (0.02 to 0.04)	<.001	<.001
Education: secondary vs primary or below	0.02 (0.00 to 0.04)	.02	.045
No debt: yes vs no^a^	0.02 (0.00 to 0.03)	.02	.04
Residence duration	0.02 (0.00 to 0.03)	.01	.03
City tier: new first tier vs others	0.01 (0.00 to 0.03)	.01	.03
Religion: yes vs no	−0.01 (−0.03 to −0.00)	.01	.03
Birth sex: others vs female^a^	−0.02 (−0.03 to −0.01)	.005	.02
Sex orientation: other vs heterosexual	−0.02 (−0.03 to −0.00)	.009	.03
Medical cost difficulty: yes vs no	−0.03 (−0.04 to −0.02)	<.001	<.001
Family type: skipped-generation family vs single-person family	−0.04 (−0.05 to −0.02)	<.001	<.001
Occupation: employed vs student	−0.04 (−0.07 to −0.02)	.001	.003
Occupation: self-employed vs student	−0.04 (−0.06 to −0.02)	<.001	<.001
Number of siblings	−0.07 (−0.08 to −0.05)	<.001	<.001
Age (years)^a^	−0.08 (−0.10 to −0.06)	<.001	<.001
Family type: couple only vs single-person family	−0.08 (−0.11 to −0.04)	<.001	<.001
Block 2: Life adversity and stress			
Socioeconomic status (youth) (1-7)	0.14 (0.13 to 0.16)	<.001	<.001
ACE^b^: psychological abuse (0‐2)	0.07 (0.05 to 0.09)	<.001	<.001
ACE: economic environment (4-20)^a^	0.06 (0.04 to 0.07)	<.001	<.001
ACE: emotional neglect: yes vs no	0.04 (0.03 to 0.06)	<.001	<.001
Life event: high study or work stress: yes vs no	0.04 (0.02 to 0.06)	<.001	<.001
Life event: serious injury or illness: yes vs no	0.03 (0.02 to 0.04)	<.001	<.001
Life event: legal dispute involvement: yes vs no	0.01 (0.00 to 0.03)	.009	.02
ACE: older brother died before 18 years of age: yes vs no	−0.01 (−0.02 to −0.00)	.007	.02
Life event: difficulty purchasing supplies: yes vs no	−0.02 (−0.04 to −0.01)	<.001	.001
Life event: other: yes vs no	−0.02 (−0.03 to −0.00)	.007	.02
ACE: collective violence exposure (0‐2)	−0.05 (-0.07 to −0.03)	<.001	<.001
ACE: physical abuse (0‐2)	−0.05 (−0.06 to −0.03)	<.001	<.001
ACE: sexual abuse (0‐4)	−0.08 (−0.10 to −0.06)	<.001	<.001
Block 3: Personality and self-efficacy			
NGSES^c^: self-efficacy (3-15)	0.10 (0.09 to 0.12)	<.001	<.001
NARQ^d^: narcissistic admiration and rivalry (6-36)	0.04 (0.03 to 0.05)	<.001	<.001
Personality (conscientiousness) (2-10)	0.03 (0.02 to 0.04)	<.001	<.001
Personality (openness) (2-10)	−0.02 (−0.03 to −0.01)	<.001	.001
Block 4: Literacy and health empowerment			
Past digital health intervention use: yes vs no	0.15 (0.14 to 0.16)	<.001	<.001
eHEALS^e^: eHealth literacy (5-25)	0.09 (0.07 to 0.10)	<.001	<.001
Family-neighbor relationship (1-7)	0.05 (0.04 to 0.07)	<.001	<.001
PSSS^f^: perceived social support (3-21)	0.04 (0.02 to 0.06)	<.001	<.001
SREBQ^g^: dietary self-regulation (5-25)	0.04 (0.03 to 0.05)	<.001	<.001
FCS-SF^h^: family communication (4-20)	0.03 (0.01 to 0.04)	<.001	<.001
Social loneliness (2-12)	−0.02 (−0.03 to −0.01)	.005	.01
Block 5: Lifestyle behaviors			
Stable sleep duration: somewhat stable vs very unstable	0.07 (0.03 to 0.10)	<.001	.001
Exposure to art: yes vs no	0.04 (0.02 to 0.05)	<.001	<.001
Sleep hours: 6‐7 hours vs ≤6 hours	0.03 (0.02 to 0.05)	<.001	<.001
Sleep hours: 7‐8 hours vs ≤6 hours	0.03 (0.01 to 0.05)	.001	.002
Daytime sleepiness: often vs never or rarely	−0.02 (−0.03 to −0.01)	.004	.01
Extra salt in food: sometimes vs never or rarely	−0.02 (−0.03 to −0.01)	.001	.003
MET^i^ category: moderate vs low	−0.02 (−0.03 to −0.01)	.004	.01
Smoking habit: yes (both) vs no	−0.02 (−0.03 to −0.00)	.01	.02
Smoking habit: yes (e-cigarettes) vs no	−0.02 (−0.03 to −0.00)	.008	.02
Chronotype: morning vs evening	−0.03 (−0.04 to −0.01)	<.001	.001
Daytime sleepiness: sometimes vs never or rarely	−0.03 (−0.04 to −0.02)	<.001	<.001
Extra salt in food: always vs never or rarely	−0.03 (−0.04 to −0.01)	<.001	<.001
Extra salt in food: often vs never or rarely	−0.03 (−0.04 to −0.02)	<.001	<.001
Sleep difficulty: sometimes vs never or rarely	−0.03 (−0.04 to −0.01)	<.001	<.001
Smoking habit: yes (conventional cigarettes) vs no	−0.03 (−0.04 to −0.02)	<.001	<.001
Block 6: Physical health and exposures			
COVID-19 vaccination: yes vs no	0.03 (0.00 to 0.05)	.02	.047
Hepatitis vaccination: yes vs no	0.02 (0.01 to 0.03)	.001	.004
Hyperlipidemia diagnosis: yes vs no	0.02 (0.01 to 0.03)	<.001	<.001
Injury event: burn or scald: yes vs no	0.02 (0.01 to 0.04)	.001	.004
Injury event: firearm injury: yes vs no	0.02 (0.01 to 0.03)	.001	.003
Arthritis diagnosis: yes vs no	−0.02 (−0.03 to −0.01)	.001	.003
COVID-19–positive count	−0.02 (−0.03 to −0.01)	<.001	.001
Flu vaccination: yes vs no	−0.02 (−0.03 to −0.00)	.01	.03
Block 7: Mental health and psychosocial			
Personal existence (1-7)	0.06 (0.04 to 0.07)	<.001	<.001
BSMAS^j^: social media addiction (6-30)	0.05 (0.04 to 0.06)	<.001	<.001
CCBI-7^k^: work burnout (7-35)	0.05 (0.04 to 0.07)	<.001	<.001
SCS^l^: cyberchondria (4-20)	0.03 (0.01 to 0.04)	<.001	.001
PHQ-9^m^: depression (0‐27)	−0.04 (−0.06 to −0.02)	.001	.002
ASRS-6^n^: ADHD^o^ symptoms (0‐24)	−0.07 (−0.09 to −0.05)	<.001	<.001

a Variables not retained by the elastic net validation.

b ACE: adverse childhood experience.

c NGSES: New General Self‑Efficacy Scale.

d NARQ: Narcissistic Admiration and Rivalry Questionnaire.

e eHEALS: eHealth Literacy Scale.

f PSSS: Perceived Social Support Scale.

g SREBQ: Self-Regulation Eating Behavior Questionnaire.

h FCS-SF: Family Communication Scale–Short Form.

i MET: metabolic equivalent of task.

j BSMAS: Bergen Social Media Addiction Scale.

k CCBI-7: 7-item Chinese version of Copenhagen Burnout Inventory.

l SCS: Short Cyberchondria Scale.

m PHQ-9: 9-item Patient Health Questionnaire.

n ASRS-6: 6-item Adult ADHD Self-Report Scale.

o ADHD: attention-deficit/hyperactivity disorder.

#### Demographics and Socioeconomics

The largest positive predictor was higher social status (β_std=0.17, 95% CI 0.16-0.18). Other positive factors included having “2 Medicare,” “only resident” Medicare, or “more than 2 Medicare”; and “graduate,” “undergraduate,” and larger “house area” (β_std=0.04-0.07). Strong negative predictors were “age” and “couple-only” family type (both β_std=−0.08). Other negative factors included “number of siblings” (β_std=−0.07, 95% CI −0.08 to −0.05), followed by “number of siblings,” “skipped-generation family” type, “employed,” and “self-employed” (β_std=−0.04 to −0.07).

#### Life Adversity and Stress

Youth socioeconomic status was the strongest positive predictor (β_std=0.14, 95% CI 0.13-0.16). Other positive predictors were “psychological abuse,” “better economic environment,” “emotional neglect,” “high study or work stress,” and “serious injury or illness” (β_std=0.03-0.07). The strongest negative predictors were “sexual abuse,” “physical abuse,” and “collective violence exposure” (β_std=−0.05 to −0.08).

#### Personality and Self-Efficacy

Self-efficacy was the most prominent predictor (β_std=0.10, 95% CI 0.09-0.12), followed by “narcissistic admiration and rivalry” (β_std=0.04, 95% CI 0.03-0.05) and “conscientiousness” (β_std=0.03, 95% CI 0.02-0.04).

#### Literacy and Health Empowerment

“Past digital health intervention use” (β_std=0.15, 95% CI 0.14-0.16) and “eHealth literacy” (β_std=0.09, 95% CI 0.07-0.10) were the strongest positive predictors. Other positive predictors included “family-neighbor relationship,” “perceived social support,” “family communication,” and “dietary self-regulation” (β_std=0.03-0.05).

#### Lifestyle Behaviors

“Stable sleep duration” (somewhat stable vs very unstable: β_std=0.07, 95% CI 0.03-0.10) was the strongest positive predictor, followed by “exposure to art,” “6‐7 hours sleep,” and “7‐8 hours sleep” (β_std=0.03-0.04). Negative predictors included “smoking habit,” “daytime sleepiness,” “extra salt in food,” “morning chronotype,” and “sleep difficulty” (all β_std=−0.03).

#### Physical Health and Exposures

“COVID-19 vaccination” (β_std=0.03, 95% CI 0.00-0.05) was the only predictor in this domain to meet the reporting threshold. No other predictors within this block were statistically significant.

#### Mental Health and Psychosocial

Positive predictors included “personal existence,” “work burnout” (β_std=0.05, 95% CI 0.04-0.07), “social media addiction,” and “cyberchondria” (β_std=0.03‐0.06). Negative predictors were “attention-deficit/hyperactivity disorder symptoms” (β_std*=*−0.07, 95% CI −0.09 to −0.05) and “depression” (β_std=−0.04, 95% CI −0.06 to −0.02).

### Classification-Tree Analysis of Acceptance

**Figure 5.**
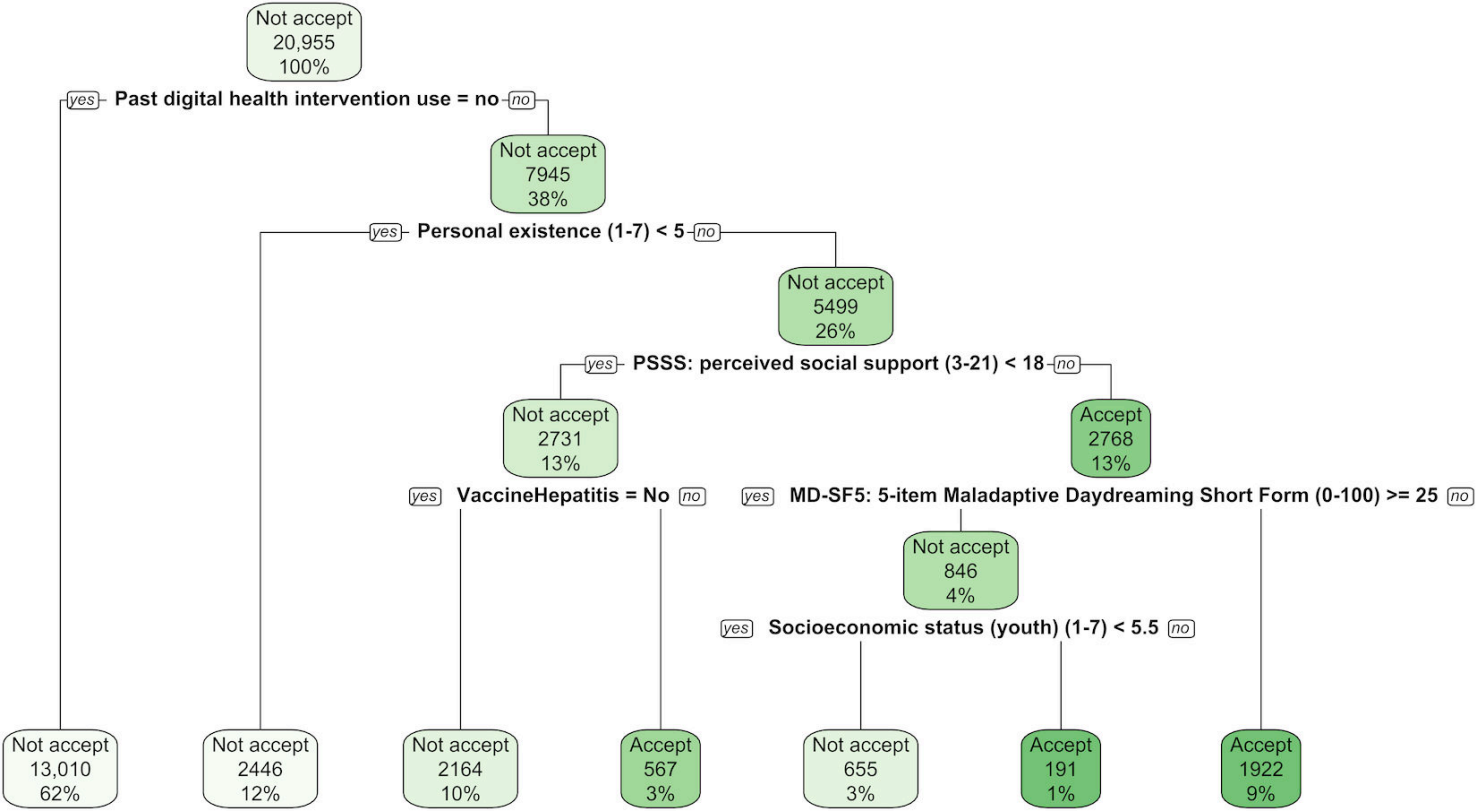
Classification decision tree model of weighted immersive extended reality acceptance in health care among adults in China, fitted on the training set from a nationwide cross-sectional survey conducted between June and September 2024 (N=20,955). MD-SF5: 5-item Maladaptive Daydreaming Short Form; PSSS: Perceived Social Support Scale.

A complete-case subsample (N=29,333) was used. The decision tree, trained on 70% (n=20,995) of the sample (Figure 5), identified 3 pathways for “acceptor” (score ≥80) and 4 pathways for “nonacceptor” (score <80). On the test set (n=8338), the tree achieved a modest weighted area under the curve of 0.61 (95% CI 0.60‐0.62), indicating modest discrimination (Figure S1 in Multimedia Appendix 1). Key performance metrics were accuracy=0.681, sensitivity=0.242 for acceptors, and specificity=0.903 for nonacceptors. Thus, the tree had limited ability to identify acceptors but performed substantially better in identifying likely nonacceptors.

The 3 “acceptor” profiles all required past digital health use and high personal existence (≥5). The largest (1922/20,955, 9%) also had high social support (Perceived Social Support Scale score ≥18) and low maladaptive daydreaming (5-item Maladaptive Daydreaming Short Form [MD-SF5] <25). The second (567/20,955, 3%) had low social support (<18) and hepatitis B vaccination. The smallest (191/20,955, 1%) had high social support, high MDS5 (≥25), and high youth socioeconomic status (≥5.5).

Conversely, 4 “nonacceptor” profiles were identified. The largest (13,010/20,955, 62% of sample) was defined simply by no past digital health use. The other 3 profiles all had past digital use but were defined by low personal existence (<5) (2446/20,955, 12%); or high personal existence, low social support (<18), and no hepatitis B vaccination (2164/20,955, 10%); or high personal existence, high social support, high MDS5 (≥25), and low youth socioeconomic status (<5.5) (655/20,955, 3%).

## Discussion

### Main Findings

This national survey addressed three aims: (1) estimating population-level acceptance of immersive XR in health care, (2) examining multidomain correlates of acceptance, and (3) developing a parsimonious tool for identifying likely nonacceptors. In relation to these aims, our findings suggest that public acceptance of immersive XR in China is moderate but uneven, with variation across geographic, demographic, and health-related subgroups. Consistent with our hypothesis, acceptance was associated not only with demographic and disease-related characteristics but also with structural, psychosocial, digital, lifestyle, and health-related factors. To facilitate interpretation, we synthesize the seven prespecified analytic blocks into four broader dimensions shaping acceptance: (1) structural-environmental factors, encompassing sociodemographic conditions and life adversity; (2) trait-resilience factors, encompassing personality, self-efficacy, and mental health; (3) social-digital capital, encompassing health literacy, empowerment, social support, and prior digital health experience; and (4) behavioral health profiles, encompassing lifestyle and physical health factors. Finally, the classification tree further suggested that a small set of variables may help identify groups with lower acceptance, although its performance indicates that it should be interpreted as a pragmatic triage aid rather than an individual-level prediction model.

### Findings in Context

Overall acceptance was modest, and our findings may provide a useful benchmark for immersive XR pilot programs before wider-scale implementation. We found that acceptance was higher in affluent coastal cities and lower with increasing age. However, even among older adults, acceptance is still influenced by prior exposure, onboarding needs, sensory and cognitive demands, and the availability of caregiver or staff support [26]. This may underscore the need to co-design age-friendly XR systems and highlights the value of future subgroup analyses within older adult populations [27]. For older adults, simplified interfaces, structured orientation, self-paced interaction, and caregiver or staff support may be especially important [26,28]. We also observed heterogeneity by health status, suggesting that acceptance is not uniform across clinical groups. This may imply that immersive XR implementation should be condition-sensitive rather than one-size-fits-all [10].

Structural and environmental factors were important correlates of acceptance. Our findings indicate that higher socioeconomic status, education, perceived social standing, and better insurance coverage were associated with higher acceptance, whereas older age, lower youth socioeconomic conditions, and some family structure indicators were associated with lower acceptance. These patterns are consistent with the possibility that digital access, cumulative social advantage, and resource constraints may shape receptivity to new health care technologies [29-35]. Notably, the positive association of psychological adverse childhood experiences (abuse and neglect) and recent stressors with higher acceptance might reflect unmeasured confounding rather than a true effect of adversity; these findings should therefore be interpreted cautiously. In contrast, sexual or physical abuse and collective adversity were linked to lower acceptance, plausibly by undermining trust and self-efficacy [36]. From a structural-environmental perspective, equitable implementation of immersive XR requires attention to digital infrastructure, affordability, and shared access models, particularly in underserved settings [27].

Acceptance was also correlated with individual traits. We found that self-efficacy was a strong positive predictor, consistent with the possibility that perceived confidence and capability facilitate receptivity to immersive health tools [25]. Newly identified correlates included conscientiousness, narcissistic admiration, burnout, social media addiction, cyberchondria, attention-deficit/hyperactivity disorder traits, and depressive symptoms. The positive associations of narcissistic admiration, burnout, social media addiction, and cyberchondria are less straightforward and should be interpreted cautiously; they may reflect greater digital immersion or health information seeking rather than inherently greater readiness for sustained XR use [37,38]. Further research is needed to determine whether these factors predict actual XR uptake and sustained engagement, or whether they are markers of broader digital engagement and health information seeking. From an implementation perspective, the strong association with self-efficacy suggests that acceptance may depend partly on users’ confidence in trying, navigating, and troubleshooting unfamiliar digital tools. Users with lower confidence or fewer social resources may therefore require more intensive guidance and follow-up [28], such as confidence-building orientation, supervised first use, and simple step-by-step instructions.

Social and digital capital also showed strong associations. Prior digital health use and higher eHealth literacy were among the strongest predictors, consistent with established adoption models, such as the Unified Theory of Acceptance and Use of Technology [39,40]. Stronger family or neighbor ties and perceived social support likely act as motivational and practical resources (eg, encouragement and troubleshooting) [40]. This may be especially true in China’s collectivist context, where endorsement from close others functions as a powerful social norm [41]. Finally, dietary self-regulation may proxy a domain-general self-regulatory capacity, which is known to predict adherence to digital health tools [42]. From an implementation perspective, the associations with eHealth literacy, prior digital health use, and social support suggest that some users may need more than access to XR equipment. Implementation may require structured onboarding, hands-on demonstrations, plain-language instructions, troubleshooting support, and optional family or caregiver involvement [9]. For example, health systems could incorporate a brief digital health literacy or digital health readiness screener into intake workflows to identify low-readiness patients for staff-assisted XR orientation, troubleshooting, and follow-up support [43,44].

To our knowledge, few prior XR acceptance studies have examined lifestyle-related factors. Our findings therefore extend previous acceptance research by suggesting that behavioral health profiles may provide additional insight into population-level acceptance of immersive XR. Specifically, we found that more stable and sufficient sleep, arts exposure, evening chronotype, and COVID-19 vaccination were associated with higher acceptance, whereas smoking and salty diet were associated with lower acceptance. These patterns may reflect broader differences in health orientation, novelty receptivity, and familiarity with digital tools [45,46]. For instance, vaccination uptake is often associated with greater institutional trust and health engagement [47]. However, these associations should be interpreted as hypothesis-generating rather than causal, and future studies should examine whether lifestyle-related profiles predict actual XR uptake, adherence, and sustained use. In terms of implementation, strategies should account for users’ behavioral health profiles and focus on tailoring interventions to these specific lifestyle factors [48], such as incorporating more motivational framing into VR design for those who smoke.

The classification tree should be interpreted as a pragmatic implementation triage aid, rather than as an individual-level predictive classifier. According to standard interpretation of classification performance metrics [49], its low sensitivity indicates limited ability to identify individuals likely to accept immersive XR. However, its high specificity suggests better performance in identifying participants unlikely to accept immersive XR. Therefore, the tree may be more useful for resource allocation and targeted outreach. In this study, the tree used 6 implementation-relevant variables: past digital health intervention use, perceived meaning in life, perceived social support, hepatitis vaccination status, childhood socioeconomic status, and MDS5. These variables may help implementation teams identify subgroups less likely to accept immersive XR in health care and proactively allocate educational resources and intensive onboarding support to those who require the most assistance [50]. XR onboarding may include prebriefing, written and verbal controller instructions, a supervised first exposure, seated low-intensity familiarization, and real-time language or technical support [51].

### Limitations

This study has several limitations. First, its cross-sectional design captures acceptance at a single time point, precluding causal inference or trend analysis. Second, all measures were self-reported and subject to recall and social desirability biases. Third, as noted, the classification-tree model performed poorly on the test set, with very low sensitivity for identifying acceptors. Fourth, the vast regional disparities observed suggest that these individual-level predictors are moderated by powerful cultural, political, or infrastructural factors that were not fully captured in this model. Therefore, our China-based sample may limit generalizability to other cultural or health system contexts. Fifth, by focusing solely on user-side determinants, this study did not examine interactions with technology-side factors, which are likely to affect uptake and warrant further investigation. Sixth, although the survey used a nationwide multistage framework, participant recruitment at the final stage was guided by quota sampling rather than a fully probability-based design. In addition, poststratification weights were calibrated only to the age-sex distribution, so residual imbalance in other characteristics, such as province, urbanicity, or education, may remain. Moreover, clustering and stratification were not incorporated into variance estimation because the necessary design information was unavailable. Seventh, the geographic analysis was strictly descriptive and did not examine the ecological drivers underlying regional variation. Future research should build on these findings by using multilevel modeling to incorporate province-level covariates to distinguish individual-level determinants from broader contextual effects.

### Conclusions

To our knowledge, this is among the first large-scale national studies to examine public acceptance of immersive health care technology using a multidomain framework in China. Unlike prior smaller or population-specific studies, it provides population-level evidence and suggests that receptivity is associated not only with demographic factors but also with structural, psychological, social, digital, behavioral, and health-related characteristics. This study contributes an equity-oriented and implementation-relevant perspective on which population groups may be more or less likely to accept immersive XR in health care. The findings suggest that equitable adoption may require more than technological availability alone; it may also depend on age-friendly design, condition-sensitive implementation, and targeted support for users with lower self-efficacy, lower digital readiness, or fewer social resources. In addition, the 6-item classification tree may offer a pragmatic starting point for identifying likely nonacceptors for outreach and support prioritization, but it requires external validation before practical use. Overall, our findings suggest that successful and equitable implementation of immersive XR is not only a technical issue but also a social, behavioral, and implementation challenge.

## Supplementary material

10.2196/88000 Multimedia Appendix 1 Supplementary materials including the receiver operating characteristic curve for the classification and regression tree model; predictor definitions and reliability metrics; weighted descriptive results by province, age group, gender, and health condition; and hierarchical weighted linear regression results across 7 predictor blocks.

## Abbreviations:

AR: augmented reality
β_std: standardized β coefficient
FDR: false discovery rate
MD-SF5: 5-item Maladaptive Daydreaming Short Form
MR: mixed reality
VR: virtual reality
XR: extended reality

## References

[1] Kafes M, Ileri YY (2025). Current status of virtual reality researches at healthcare: thematic and bibliometric analysis. Front Virtual Real.

[2] Iqbal AI, Aamir A, Hammad A (2024). Immersive technologies in healthcare: an in-depth exploration of virtual reality and augmented reality in enhancing patient care, medical education, and training paradigms. J Prim Care Community Health.

[3] Faizan Siddiqui M, Jabeen S, Alwazzan A (2025). Integration of augmented reality, virtual reality, and extended reality in healthcare and medical education: a glimpse into the emerging horizon in LMICs—a systematic review. J Med Educ Curric Dev.

[4] Arthur T, Melendez-Torres GJ, Harris D, Robinson S, Wilson M, Vine S (2025). Extended reality interventions for health and procedural anxiety: panoramic meta-analysis based on overviews of reviews. J Med Internet Res.

[5] Tang P, Cao Y, Vithran DTAV (2025). The efficacy of virtual reality on the rehabilitation of musculoskeletal diseases: umbrella review. J Med Internet Res.

[6] Fereidooni M, Toni E, Toni E, Ayatollahi H (2024). Application of virtual reality for supportive care in cancer patients: a systematic review. Support Care Cancer.

[7] Hao J, Crum G, Siu KC (2024). Effects of virtual reality on stroke rehabilitation: an umbrella review of systematic reviews. Health Sci Rep.

[8] Sung H, Kim M, Park J, Shin N, Han Y (2024). Effectiveness of virtual reality in healthcare education: systematic review and meta-analysis. Sustainability.

[9] Kouijzer MMTE, Kip H, Bouman YHA, Kelders SM (2023). Implementation of virtual reality in healthcare: a scoping review on the implementation process of virtual reality in various healthcare settings. Implement Sci Commun.

[10] Elser A, Lange M, Kopkow C, Schäfer AG (2024). Barriers and facilitators to the implementation of virtual reality interventions for people with chronic pain: scoping review. JMIR XR Spat Comput.

[11] Arthur T, Robinson S, Vine S, Asare L, Melendez-Torres GJ (2025). Equity implications of extended reality technologies for health and procedural anxiety: a systematic review and implementation-focused framework. J Am Med Inform Assoc.

[12] Wang EY, Qian D, Zhang L (2024). Acceptance of virtual reality in trainees using a technology acceptance model: survey study. JMIR Med Educ.

[13] Felsberg DT, McGuirt JT, Ross SE, Raisbeck LD, Howard CK, Rhea CK (2025). Clinician perspectives on virtual reality use in physical therapy practice in the United States. PLoS One.

[14] Raghuraman N, Bedford T, Tran N, Haycock NR, Wang Y, Colloca L (2024). The interplay between health disparities and acceptability of virtual reality: a survey study. Cyberpsychol Behav Soc Netw.

[15] Zeng F, Li Q, Cai S (2025). Cancer patients’ acceptance of virtual reality interventions for self-emotion regulation. Sci Rep.

[16] Chen Y, Guan B, Zhang Y (2025). Acceptability of and willingness to use virtual reality exergames for weight loss among young adults with overweight or obesity in China: qualitative study. JMIR Serious Games.

[17] Sauchelli S, Pickles T, Voinescu A (2023). Public attitudes towards the use of novel technologies in their future healthcare: a UK survey. BMC Med Inform Decis Mak.

[18] Huygelier H, Schraepen B, van Ee R, Vanden Abeele V, Gillebert CR (2019). Acceptance of immersive head-mounted virtual reality in older adults. Sci Rep.

[19] Moore RC, Hancock JT, Bailenson JN (2023). From 65 to 103, older adults experience virtual reality differently depending on their age: evidence from a large-scale field study in nursing homes and assisted living facilities. Cyberpsychol Behav Soc Netw.

[20] Keller MS, Park HJ, Cunningham ME, Fouladian JE, Chen M, Spiegel BMR (2017). Public perceptions regarding use of virtual reality in health care: a social media content analysis using Facebook. J Med Internet Res.

[21] Dy M, Olazo K, Lisker S (2023). Virtual reality for chronic pain management among historically marginalized populations: systematic review of usability studies. J Med Internet Res.

[22] Siette J, Adam PJ, Harris CB (2024). Acceptability of virtual reality to screen for dementia in older adults. BMC Geriatr.

[23] Wong EYC, Hui RTY, Kong H (2023). Perceived usefulness of, engagement with, and effectiveness of virtual reality environments in learning industrial operations: the moderating role of openness to experience. Virtual Real.

[24] Al Khalifah E, Hammady R, Abdelrahman M (2025). Technology anxiety in virtual reality adoption: examining the impact of age, past experience, and cybersickness. IEEE Access.

[25] Liu Y, Lu X, Zhao G, Li C, Shi J (2022). Adoption of mobile health services using the unified theory of acceptance and use of technology model: self-efficacy and privacy concerns. Front Psychol.

[26] Schaumburg M, Imtiaz A, Zhou R, Bernard M, Wolbers T, Segen V (2025). Immersive virtual reality for older adults: challenges and solutions in basic research and clinical applications. Ageing Res Rev.

[27] Richardson S, Lawrence K, Schoenthaler AM, Mann D (2022). A framework for digital health equity. NPJ Digit Med.

[28] Li Y, Shiyanov I, Muschalla B (2024). Older adults’ acceptance of a virtual reality group intervention in nursing homes: pre-post study under naturalistic conditions. JMIR Hum Factors.

[29] Tuitert I, Marinus JD, Dalenberg JR, van ’t Veer JT (2024). Digital health technology use across socioeconomic groups prior to and during the COVID-19 pandemic: panel study. JMIR Public Health Surveill.

[30] Li Q, Cheng F, Zeng H, Xu J (2024). Health insurance payment for telehealth services: scoping review and narrative synthesis. J Med Internet Res.

[31] Montagni I, Cariou T, Feuillet T, Langlois E, Tzourio C (2018). Exploring digital health use and opinions of university students: field survey study. JMIR Mhealth Uhealth.

[32] Bertolazzi A, Quaglia V, Bongelli R (2024). Barriers and facilitators to health technology adoption by older adults with chronic diseases: an integrative systematic review. BMC Public Health.

[33] Zainal H, Xiaohui X, Thumboo J, Seah SJ, Leng LL, Kok Yong F (2025). Exploring caregiver challenges, digital health technologies, and healthcare support: a qualitative study. Front Digit Health.

[34] Downey DB (2001). Number of siblings and intellectual development. The resource dilution explanation. Am Psychol.

[35] Silver MP (2014). Socio-economic status over the lifecourse and internet use in older adulthood. Ageing Soc.

[36] Diehl AS, Prout MF (2002). Effects of posttraumatic stress disorder and child sexual abuse on self-efficacy development. Am J Orthopsychiatry.

[37] El-Zayat A, Namnkani SA, Alshareef NA, Mustfa MM, Eminaga NS, Algarni GA (2023). Cyberchondria and its association with smartphone addiction and electronic health literacy among a Saudi population. Saudi J Med Med Sci.

[38] Casale S, Banchi V (2020). Narcissism and problematic social media use: a systematic literature review. Addict Behav Rep.

[39] Rahimi B, Nadri H, Lotfnezhad Afshar H, Timpka T (2018). A systematic review of the technology acceptance model in health informatics. Appl Clin Inform.

[40] Shiu LS, Huang YS, Liu CY, Cheng YS, Chen YC (2026). eHealth literacy mediating social support and technology acceptance among patients with chronic illnesses: a cross-sectional study. J Adv Nurs.

[41] Venkatesh V, Zhang X (2010). Unified Theory of Acceptance and Use of Technology: U.S. Vs. China. Journal of Global Information Technology Management.

[42] Plaitano EG, McNeish D, Bartels SM (2025). Adherence to a digital therapeutic mediates the relationship between momentary self-regulation and health risk behaviors. Front Digit Health.

[43] Nelson LA, Pennings JS, Sommer EC, Popescu F, Barkin SL (2022). A 3-item measure of digital health care literacy: development and validation study. JMIR Form Res.

[44] Rising KL, Guth A, Gentsch AT (2024). Development and preliminary validation of a screener for digital health readiness. JAMA Netw Open.

[45] Kortesoja L, Vainikainen MP, Hotulainen R, Merikanto I (2023). Late-night digital media use in relation to chronotype, sleep and tiredness on school days in adolescence. J Youth Adolesc.

[46] White TM, Wyka K, Rabin K, El-Mohandes A (2024). Trust in the science behind COVID-19 vaccines as a driver of vaccine acceptance in the United States, 2021-2023. Vaccine X.

[47] Krastev S, Krajden O, Vang ZM (2023). Institutional trust is a distinct construct related to vaccine hesitancy and refusal. BMC Public Health.

[48] Noar SM, Benac CN, Harris MS (2007). Does tailoring matter? Meta-analytic review of tailored print health behavior change interventions. Psychol Bull.

[49] Pewsner D, Battaglia M, Minder C, Marx A, Bucher HC, Egger M (2004). Ruling a diagnosis in or out with “SpPIn” and “SnNOut”: a note of caution. BMJ.

[50] Kappen TH, van Klei WA, van Wolfswinkel L, Kalkman CJ, Vergouwe Y, Moons KGM (2018). Evaluating the impact of prediction models: lessons learned, challenges, and recommendations. Diagn Progn Res.

[51] Leung RYF, Ye MZ, Zhang FY (2025). Feasibility and efficacy of commercial-off-the-shelf virtual reality applications for managing chronic pain and enhancing well-being among older adults in the community: mixed methods pilot study. JMIR Form Res.

