# Supplementary material for: National Acceptance and Determinants of Immersive Extended Reality in Health Care in China: Cross-Sectional Study
Source: J Med Internet Res. 2026 Jun 26;28:e88000. doi: 10.2196/88000 (PMC13308750; doi:10.2196/88000)
Supplement: Multimedia Appendix 1 [file jmir-v28-e88000-s001.docx]

**Multimedia Appendix 1**

**Figure S1.** ROC curve for the CART model (test set), with shaded area under the curve and annotated AUC (95% bootstrap CI).

**
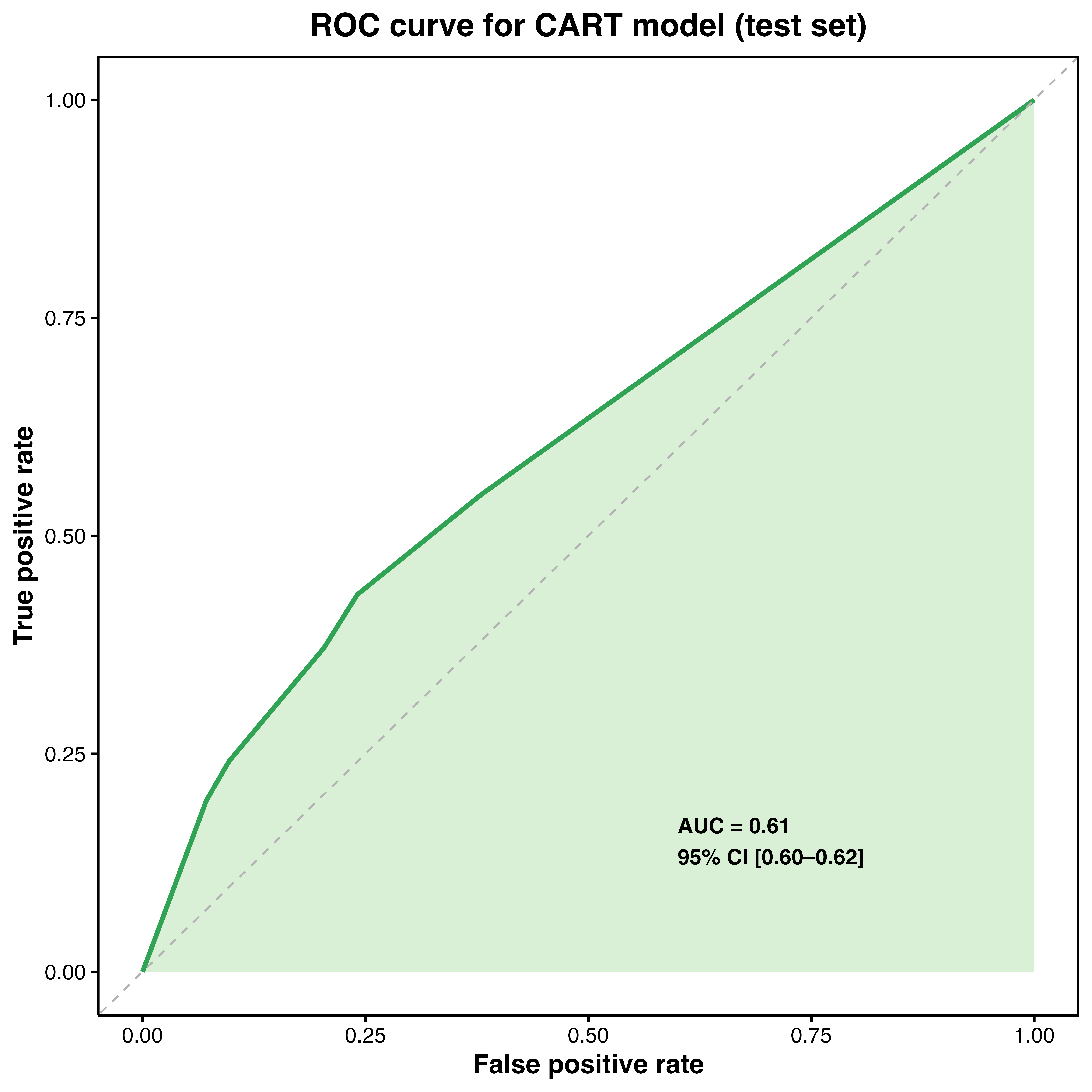
**

***Notes*:** ROC, receiver operating characteristic; CART: Classification and Regression Tree; AUC, area under the curve; CI, confidence interval.

**Table S1.** Definitions, measurement ranges, and reliability metrics for all predictor variables across seven blocks.

| **Variables** | **Measurement type (categorical or continuous), with scale description (if applicable)** |
| --- | --- |
| **Block 1: demographics and socioeconomics** |  |
| Age | Continuous (range: 18-124) |
| Birth sex | Male/Female/Others |
| Race | Han/Others |
| Relationship status | Single/In relationship/Married/Divorced/Widowed |
| Occupation | Employed/Retired/Self-employed/Student/Unemployed |
| Education | Primary or below/Secondary/Undergraduate/Graduate |
| Per-person income | Continuous (1–10 levels), where 1 = ¥≤1 000; 2 = ¥1 001–2 000; 3 = ¥2 001–3 000; 4 = ¥3 001–4 000; 5 = ¥4 001–5 000; 6 = ¥5 001–6 000; 7 = ¥6 001–9 000; 8 = ¥9 001–12 000; 9 = ¥12 001–15 000; and 10 = ¥≥15 001. |
| Religion | No/Yes |
| Handedness | Left hand/ Right hand |
| Family type | Couple only/Nuclear family/Extended family/Joint family/Single-parent family/DINK/Skipped-generation family/Single-person family/Others |
| Number of siblings | Continuous (range: 0-3) |
| Sex orientation | Heterosexual/Homosexual/Bisexual/Asexual/Others |
| Gender identity | Male/Female/Transgender male (female-to-male)/Transgender female (male-to-female)/Genderqueer or genderfluid/Others |
| Medical insurance | None/Government-funded only/Urban resident only/Employee only/New rural cooperative only/Commercial only/ Two types/ More than two types |
| Medical cost difficulty | No/Yes |
| Medicare enrollment location | Living area/Hukou location/Others |
| City tier | First tier/New first tier/Others |
| Residence duration | Continuous (1–6 levels), where 1 = six months or less; 2 = more than six months to less than one year; 3 = more than one year to less than five years; 4 = more than five years to less than ten years; 5 = ten years or more; and 6 = since birth. |
| Residence (last 3 months) | Urban/Rural |
| Household registration | Urban/Rural |
| House area | Continuous (1–6 levels), where 1 = <60 m²; 2 = ≥60 and <90 m²; 3 = ≥90 and <120 m²; 4 = ≥120 and <150 m²; 5 = ≥150 and <180 m²; and 6 = ≥180 m². |
| House structure: number of bedrooms | Continuous (0–4 levels), where 0 = no bedroom; 1 = one-bedroom; 2 = two-bedroom; 3 = three-bedroom; and 4 = four-bedroom or more. |
| Number of properties owned | Continuous (0–3 levels), where 0 = no property; 1 = one property; 2 = two properties; and 3 = three or more properties. |
| No debt | No/Yes |
| Lived alone in past 3 months | No/Yes |
| Social status | Continuous (range: 1-7), where 1=lowest; 7=highest |
| **Block 2: life adversity and stress** |  |
| Life event: immediate family death (yes vs. no) | No/Yes |
| Life event: unemployment (yes vs. no) | No/Yes |
| Life event: legal dispute involvement (yes vs. no) | No/Yes |
| Life event: high study/work stress (yes vs. no) | No/Yes |
| Life event: family economic hardship (yes vs. no) | No/Yes |
| Life event: unable to reunite with family (yes vs. no) | No/Yes |
| Life event: major change in routine (yes vs. no) | No/Yes |
| Life event: family conflict (yes vs. no) | No/Yes |
| Life event: difficulty purchasing supplies (yes vs. no) | No/Yes |
| Life event: accident or natural disaster (yes vs. no) | No/Yes |
| Life event: theft or property loss (yes vs. no) | No/Yes |
| Life event: serious injury or illness (yes vs. no) | No/Yes |
| Life event: other (yes vs. no) | No/Yes |
| Life event: none (yes vs. no) | No/Yes |
| ACE: psychological abuse (0–2) | Continuous (range: 0-2)  Scale: The Psychological Abuse subscale of the WHO Adverse Childhood Experiences (ACE‐WHO) questionnaire is a two‐item measure assessing exposure to emotional maltreatment during childhood. Designed for adults (18 years and older), respondents indicate how often they experienced each form of psychological abuse using four frequency options (usually, sometimes, rarely, never). In scoring, “usually” and “sometimes” responses each receive 1 point, while “rarely” and “never” receive 0 points. Subscale scores range from 0 to 2, with higher scores reflecting greater exposure to childhood psychological abuse.  Reference: Fan S, Li Y, Dong F, et al. Study protocol: A national cross-sectional study on psychology and behaviour investigation of Chinese residents in 2024, PBICR. medRxiv. 2025:2025.01. 14.25320520. |
| ACE: emotional neglect (yes vs. no) | No/Yes  Scale: The Emotional Neglect item is a single‐question measure from the ACE (Adverse Childhood Experiences) questionnaire that assesses whether an adult (18 years and older) experienced emotional neglect during childhood. Respondents indicate how often they felt emotionally neglected by choosing one of four options: usually, sometimes, rarely, or never. In scoring, “usually” and “sometimes” each receive 1 point, while “rarely” and “never” receive 0 points. Thus, total scores range from 0 to 1, with a score of 1 reflecting greater exposure to childhood emotional neglect.  Reference: Fan S, Li Y, Dong F, et al. Study protocol: A national cross-sectional study on psychology and behaviour investigation of Chinese residents in 2024, PBICR. medRxiv. 2025:2025.01. 14.25320520. |
| ACE: physical abuse (0–2) | Continuous (range: 0-2)  Scale: The Physical Abuse subscale of the ACE‐WHO questionnaire comprises two items assessing exposure to physical maltreatment during childhood. Adults (18+ years) indicate how often they experienced each form of physical abuse by selecting one of four options— “usually,” “sometimes,” “rarely,” or “never.” Responses of “usually” or “sometimes” are scored as 1 point, while “rarely” or “never” are scored as 0 points. Subscale scores range from 0 to 2, with higher scores reflecting greater exposure to childhood physical abuse.  Reference: Fan S, Li Y, Dong F, et al. Study protocol: A national cross-sectional study on psychology and behaviour investigation of Chinese residents in 2024, PBICR. medRxiv. 2025:2025.01. 14.25320520. |
| ACE: sexual abuse (0–4) | Continuous (range: 0-4)  Scale: The Sexual Abuse subscale of the ACE-WHO questionnaire consists of four items assessing experiences of unwanted sexual contact or coercion during childhood. Administered to adults (18 years and older), respondents indicate how often each event occurred by choosing “usually,” “sometimes,” “rarely,” or “never.” “Usually” and “sometimes” responses are each scored as 1 point, while “rarely” and “never” are scored as 0. Total scores therefore range from 0 to 4, with higher scores reflecting greater exposure to childhood sexual abuse.  Reference: Fan S, Li Y, Dong F, et al. Study protocol: A national cross-sectional study on psychology and behaviour investigation of Chinese residents in 2024, PBICR. medRxiv. 2025:2025.01. 14.25320520. |
| ACE: father died before age 18 (yes vs. no) | No/Yes |
| ACE: mother died before age 18 (yes vs. no) | No/Yes |
| ACE: older brother died before age 18 (yes vs. no) | No/Yes |
| ACE: younger brother died before age 18 (yes vs. no) | No/Yes |
| ACE: older sister died before age 18 (yes vs. no) | No/Yes |
| ACE: younger sister died before age 18 (yes vs. no) | No/Yes |
| ACE: no relative died before age 18 (yes vs. no) | No/Yes |
| ACE: witnessed maternal violence (0–4) | Continuous (range: 0-4)  Scale: The Violence Against Mother or Stepmother subscale of the ACE‐WHO questionnaire comprises four items assessing how often an adult (18+ years) witnessed or was aware of violence directed at their mother or stepmother during childhood. Respondents choose one of four frequency options— “usually,” “sometimes,” “rarely,” or “never.” “Usually” and “sometimes” responses each receive 1 point, while “rarely” and “never” receive 0 points. Scores range from 0 to 4, with higher totals reflecting greater exposure to violence against a mother or stepmother in childhood.  Reference: Fan S, Li Y, Dong F, et al. Study protocol: A national cross-sectional study on psychology and behaviour investigation of Chinese residents in 2024, PBICR. medRxiv. 2025:2025.01. 14.25320520. |
| ACE: community violence exposure (0–2) | Continuous (range: 0-2)  Scale: The Community Violence subscale of the ACE questionnaire consists of two items assessing how often, during childhood, the respondent witnessed or experienced violence in their neighborhood or community. Administered to adults (18+), each item is answered using four frequency options (“usually,” “sometimes,” “rarely,” or “never”). Responses of “usually” or “sometimes” are scored as 1 point, while “rarely” and “never” are scored as 0. Total scores therefore range from 0 to 2, with higher scores indicating greater exposure to community violence in childhood.  Reference: Fan S, Li Y, Dong F, et al. Study protocol: A national cross-sectional study on psychology and behaviour investigation of Chinese residents in 2024, PBICR. medRxiv. 2025:2025.01. 14.25320520. |
| ACE: collective violence exposure (0–2) | Continuous (range: 0-2)  Scale: The Collective Violence subscale (CE) is a two-item, self-developed measure assessing exposure to large‐scale violence during childhood. Designed for adults (18+), respondents report how often they experienced or witnessed collective violence by choosing “usually,” “sometimes,” “rarely,” or “never.” Responses of “usually” or “sometimes” are scored as 1 point each, while “rarely” and “never” receive 0 points. Subscale scores range from 0 to 2, with higher totals indicating greater exposure to collective violence in childhood.  Reference: Fan S, Li Y, Dong F, et al. Study protocol: A national cross-sectional study on psychology and behaviour investigation of Chinese residents in 2024, PBICR. medRxiv. 2025:2025.01. 14.25320520. |
| ACE: economic environment (4–20) | Continuous (range:4-10)  Scale: The Economic Environment subscale of the Childhood Experiences (CE) measure comprises four self-developed items assessing perceived family economic circumstances during childhood. Administered to adults (18+ years), respondents rate each statement on a 5-point Likert scale (1 = strongly disagree to 5 = strongly agree). Total scores range from 4 to 20, with higher scores indicating more favorable childhood family economic conditions.  Reference: Fan S, Li Y, Dong F, et al. Study protocol: A national cross-sectional study on psychology and behaviour investigation of Chinese residents in 2024, PBICR. medRxiv. 2025:2025.01. 14.25320520. |
| Socioeconomic status (youth) (1–7) | Continuous (range: 1-7), where "1"= "lowest socioeconomic status and "7"= "highest socioeconomic status |
| **Block 3: personality** |  |
| Personality (extraversion) (2–10) | Continuous(range:2-10)  Scale: The 10-item Big Five Inventory was used to measure the personality traits of the population, including extraversion, agreeableness, conscientiousness, neuroticism, and openness. The scale consists of five dimensions, each containing two entries. A five-point Likert scale ranging from 1 “strongly disagree” to 5 “strongly agree” was used. Each subscale is scored on a scale of 2–10. Higher scores represent higher levels of personality traits. The Cronbach's alpha coefficients for extraversion, agreeableness, conscientiousness, neuroticism, and openness were 0.723, 0.759, 0.786, 0.753, and 0.714, respectively.  Reference: Wang Y, Yao L, Liu L, et al. The mediating role of self-efficacy in the relationship between Big five personality and depressive symptoms among Chinese unemployed population: a cross-sectional study. BMC Psychiatry. 2014;14:61. |
| Personality (agreeableness) (2–10) |  |
| Personality (conscientiousness) (2–10) |  |
| Personality (neuroticism) (2–10) |  |
| Personality (openness) (2–10) |  |
| NGSES: self-efficacy (3–15) | Continuous (range: 3-15)  Scale: [Self-efficacy was measured using the 3-item New General Self-Efficacy Short Form. Each item, scored from 1 (“very difficult”) to 5 (“strongly agree”), assesses confidence in achieving goals across different contexts. Higher scores indicate greater self-efficacy. The Chinese version demonstrated excellent psychometric properties (Cronbach α=0.94), whereas our sample showed a Cronbach α of 0.89.](https://www.jmir.org/2025/1/e70822#ref37)  Reference: Wang F, Chen K, Du Z, Wu Y, Tang J, Sun X. Reliability and validity analysis and Mokken model of New General Self-Efficacy Scale-Short Form (NGSES-SF). 2022. |
| NARQ: narcissistic admiration & rivalry (6–36) | Continuous (range: 6-36)  Scale: The six‐item short form of the Narcissistic Admiration and Rivalry Questionnaire (NARQ-6) assesses both agentic (admiration) and antagonistic (rivalry) facets of grandiose narcissism. Respondents rate each item (e.g., “I want my rivals to fail.”) on a 6‐point Likert scale (1 = do not agree at all to 6 = agree completely). Total scores range from 6 to 36, with higher values indicating greater narcissistic admiration and rivalry. In its validation, internal consistency was acceptable (Cronbach’s α = .74).  Reference: Leckelt M, Wetzel E, Gerlach TM, et al. Validation of the Narcissistic Admiration and Rivalry Questionnaire Short Scale (NARQ-S) in convenience and representative samples. Psychological assessment. 2018;30(1):86. |
| **Block 4: literacy and health empowerment** |  |
| Family-neighbor relationship (1–7) | Continuous (range: 1-7), where 1=very poor; 7=very good |
| Have someone to trust: no vs. yes | No/Yes |
| Family doctor contract: no vs. yes | No/Yes |
| eHEALS: ehealth literacy (5–25) | Continuous (range: 5-25)  Scale: Health empowerment outcomes included eHealth literacy, perceived social support, and self-efficacy. eHealth literacy was assessed using the 5-item Chinese version of the eHealth Literacy Scale (eHEALS). Each item was rated on a Likert scale from 1 (strongly disagree), 3 (unsure), to 5 (strongly agree), resulting in total scores ranging from 5 to 25. Higher scores indicate greater eHealth literacy. The Chinese version of the eHEALS demonstrated excellent reliability, with a Cronbach α of 0.95, and our sample confirmed similar reliability with a Cronbach α of 0.94.  Reference: Li J, Wu X, Wu Y, et al. Physical, Mental, and Health Empowerment Disparities Across Chronic Obstructive Pulmonary Disease, Asthma, and Combined Groups and the Moderating Role of eHealth Literacy: Cross-Sectional Study. Journal of medical Internet research. 2025;27:e70822. |
| FHS-SF: family health (10–50) | Continuous (range: 10-50)  The FHS-SF is a short version of the Family Health Scale (FHS) that measures family health functioning. The FHS-SF consists of 10 items in 4 dimensions: internal family emotional communication, family healthy lifestyle, family health resources, and external social support, all of which are scored on a 5-point Likert scale. The total score of the scale is 10, with 0–5 as unhealthy families, 6–8 as generally healthy families, and 9–10 as healthy families. The Cronbach's alpha coefficient was 0.86.  Reference: Crandall A, Weiss-Laxer NS, Broadbent E, et al. The family health scale: reliability and validity of a short-and long-form. Frontiers in Public Health. 2020;8:587125. |
| HLS-SF: health literacy (0–12) | Continuous (range: 0-12)  Scale: The HLS-SF contains four items. Each item is scored on a 4-point scale from 0 (very difficult) to 3 (very easy). The summed items have a total score from 0 to 12, with a higher score demonstrating better health literacy. The Cronbach's alpha coefficient was 0.84.  Reference: SUN X, CHEN K, WU Y, et al. Development of a short version of the health literacy scale based on classical test theory and item response theory. Chinese General Practice. 2024;27(23):2931. |
| Received help when needed: no vs. yes | No/Yes |
| Social loneliness (2–12) | Continuous (range:2-12)  Scale: The Social Loneliness Indicators (SLI) scale is a two‐item self‐report measure of perceived loneliness. Respondents answer “How often do you feel lonely?” and “How often do you confide in a family member or friend?” using a 6‐point frequency scale (1 = rarely or never; 2 = once every three months; 3 = once a month; 4 = once a week; 5 = two to four times a week; 6 = daily). To compute a single score where higher values indicate less loneliness, reverse‐code the “feel lonely” item (so that 6 = never and 1 = daily) and leave the “confide” item unchanged; then sum both items. Total scores range from 2 to 12, with higher totals reflecting lower levels of social loneliness.  Reference: Fan S, Li Y, Dong F, et al. Study protocol: A national cross-sectional study on psychology and behaviour investigation of Chinese residents in 2024, PBICR. medRxiv. 2025:2025.01. 14.25320520. |
| Social isolation (3–18) | Continuous(range:0-3)  Scale: The Social Isolation Indicators (SII) scale comprises three items asking adults (18+) how often they (1) contact family or friends by phone, (2) have family or friends visit, and (3) visit family or friends. Each item is rated on a 6-point frequency scale (1 = rarely or never; 2 = once every three months; 3 = once a month; 4 = once a week; 5 = two to four times a week; 6 = daily). For scoring, each response is reverse-coded (so that 1→6, 2→5, 3→4, 4→3, 5→2, 6→1), and the three reversed values are summed. Total scores range from 3 to 18, with higher totals indicating greater social isolation.  Reference: Fan S, Li Y, Dong F, et al. Study protocol: A national cross-sectional study on psychology and behaviour investigation of Chinese residents in 2024, PBICR. medRxiv. 2025:2025.01. 14.25320520. |
| Social connection (5–30) | Continuous(range:0-5)  Scale: The Social Connection Index (SCI) comprises five items that ask how often, over the past period, they (1) go to the cinema or attend sporting events, (2) travel, (3) participate in board games or similar recreational activities, (4) engage in activities at bars, clubs, sports associations, or gyms, and (5) attend religious groups, adult education classes, or other group events. For each item, respondents choose one of six frequency options: 1 = rarely or never; 2 = once every three months; 3 = once a month; 4 = once a week; 5 = two to four times a week; 6 = daily. To compute a total SCI score (range: 5–30), sum the raw item values—higher totals reflect greater social connectedness.  Reference: Fan S, Li Y, Dong F, et al. Study protocol: A national cross-sectional study on psychology and behaviour investigation of Chinese residents in 2024, PBICR. medRxiv. 2025:2025.01. 14.25320520. |
| PSSS: perceived social support (3–21) | Continuous (range: 3-21)  Scale: [Perceived social support was measured using the perceived social support scale, a 3-item instrument rated on a 7-point Likert scale ranging from “extremely disagree” to “extremely agree.” Total scores ranged from 3 to 21, with higher scores indicating a greater level of perceived social support. This scale assessed support from family, friends, and others and demonstrated high internal consistency, with a Cronbach α of 0.89. In our sample, the Cronbach α was 0.86.](https://www.jmir.org/2025/1/e70822#ref36)  Reference: Wang F, Wu Y, Sun X, et al. Reliability and validity of the Chinese version of a short form of the family health scale. BMC primary care. 2022;23(1):108. |
| Implicit health beliefs (6–36) | Continuous (range: 6-36)  Scale: The Implicit Health Beliefs Scale is a six‐item psychological measure designed for adults to assess how individuals conceptualize health—either as something that can progressively change or as a fixed entity. Each item taps one of two dimensions (progressive lay health theory versus entity lay health theory), and respondents rate their agreement on a 6‐point Likert scale (1 = strongly disagree to 6 = strongly agree). By summing all item responses, total scores range from 6 to 36, with higher scores indicating a stronger endorsement of incremental (progressive) views of health relative to a fixed (entity) perspective. This scale is applicable across all adult populations.  Reference: Fan S, Li Y, Dong F, et al. Study protocol: A national cross-sectional study on psychology and behaviour investigation of Chinese residents in 2024, PBICR. medRxiv. 2025:2025.01. 14.25320520. |
| FCS-SF: family communication (4–20) | Continuous (range: 4-20)  Scale: The simplified 4-item Family Communication Scale is a measure of positive family communication. Each statement is rated on a 5-point Likert scale (1 = strongly disagree to 5 = strongly agree), yielding a total score range of 4–20. Higher scores indicate better family communication. In this study, the scale demonstrated excellent internal consistency (Cronbach’s α = 0.92).  Reference: Fan S, Li Y, Dong F, et al. Study protocol: A national cross-sectional study on psychology and behaviour investigation of Chinese residents in 2024, PBICR. medRxiv. 2025:2025.01. 14.25320520. |
| SREBQ: dietary self-regulation (5–25) | Continuous (range: 5-25)  Scale: The Self-Regulation of Eating Behavior Questionnaire (SREBQ) is a five-item measure of an individual’s capacity to self-regulate eating behaviors. Respondents rate each statement on a 5-point Likert scale (1 = Never to 5 = Always), with total scores ranging from 5 to 25—higher scores reflect stronger self-regulation. The SREBQ demonstrated good internal consistency (Cronbach α = 0.75)  Reference: Kliemann N, Beeken RJ, Wardle J, Johnson F. Development and validation of the self-regulation of eating behaviour questionnaire for adults. International Journal of Behavioral Nutrition and Physical Activity. 2016;13:1-11. |
| Antibiotic knowledge: no vs. yes | Yes/No |
| Past digital health intervention use (yes vs no) | No/Yes |
| **Block 5: lifestyle behaviors** |  |
| Smoking habit | No/Conventional/E-cigarettes/Conventional + e-cigarettes/Former smoker |
| Alcohol use | Never drink/Current frinker/Former drinker/New drinker |
| MET category | Low/Moderate/high  Scale: Physical activity levels were assessed using the International Physical Activity Questionnaire-7. Basal metabolic time per week was calculated by summing the metabolic equivalents (METs) for activities of varying intensities: mild-intensity (3.3 METs), moderate-intensity (4.0 METs), and strenuous-intensity (8.0 METs). For each activity level, METs were multiplied by the average daily duration and the number of days per week, and total weekly MET minutes were calculated. Physical activity was classified as low (< 600 MET-min/week), moderate (≥ 600 to < 3,000 MET-min/week), or high (≥ 3,000 MET-min/week).  Reference: Liou YM, Jwo CJ, Yao KG, Chiang L-C, Huang L-H. Selection of appropriate Chinese terms to represent intensity and types of physical activity terms for use in the Taiwan version of IPAQ. Journal of Nursing Research. 2008;16(4):252-263. |
| Chronotype | Morning/Evening |
| Sleep hours | ≤ 6 hours/6-7 hours/7-8 hours/8-9 hours/>9 |
| Sleep difficulty | Never or rarely/Sometimes/Often |
| Snore | No/Yes |
| Daytime sleepiness | Never or rarely/Sometimes/Often/Always |
| Stable sleep duration | Very unstable/Somewhat unstable/Somewhat stable/Very stable |
| Extra salt in food | Never or rarely/Sometimes/Often/Always |
| Exposure to music: yes vs. no | No/Yes |
| Exposure to art: yes vs. no | No/Yes |
| Exposure to dance: yes vs. no | No/Yes |
| Exposure to other arts: yes vs. no | No/Yes |
| **Block 6: physical health and exposures** |  |
| BMI classification (ref: severely underweight) | Severely underweight/Underweight/Normal weight/Overweight/Obesity |
| Hypertension diagnosis | No/Yes |
| Diabetes diagnosis (yes vs. no) | No/Yes |
| Hyperlipidemia diagnosis (yes vs. no) | No/Yes |
| Coronary heart disease diagnosis (yes vs. no) | No/Yes |
| Stroke diagnosis (yes vs. no) | No/Yes |
| Respiratory disease diagnosis (yes vs. no) | No/Yes |
| Urinary disease diagnosis (yes vs. no) | No/Yes |
| Digestive disease diagnosis (yes vs. no) | No/Yes |
| Osteoporosis diagnosis (yes vs. no) | No/Yes |
| Arthritis diagnosis (yes vs. no) | No/Yes |
| Tumor diagnosis (yes vs. no) | No/Yes |
| Rare disease diagnosis (yes vs. no) | No/Yes |
| Other diagnosis (yes vs. no) | No/Yes |
| No diagnosis (yes vs. no) | No/Yes |
| Injury event: motor vehicle accident (yes vs. no) | No/Yes |
| Injury event: non‐motor vehicle accident (yes vs. no) | No/Yes |
| Injury event: fall or falling (yes vs. no) | No/Yes |
| Injury event: blunt instrument injury (yes vs. no) | No/Yes |
| Injury event: firearm injury (yes vs. no) | No/Yes |
| Injury event: sharp‐object injury (yes vs. no) | No/Yes |
| Injury event: burn or scald (yes vs. no) | No/Yes |
| Injury event: suffocation or hanging (yes vs. no) | No/Yes |
| Injury event: drowning (yes vs. no) | No/Yes |
| Injury event: poisoning (yes vs. no) | No/Yes |
| Injury event: animal‐related injury (yes vs. no) | No/Yes |
| Injury event: sexual assault (yes vs. no) | No/Yes |
| Injury event: other (yes vs. no) | No/Yes |
| Injury event: none (yes vs. no) | No/Yes |
| HPV vaccination (yes vs. no) | No/Yes |
| Flu vaccination (yes vs. no) | No/Yes |
| Shingles vaccination (yes vs. no) | No/Yes |
| Hepatitis vaccination (yes vs. no) | No/Yes |
| COVID vaccination (yes vs. no) | No/Yes |
| No vaccination (yes vs. no) | No/Yes |
| COVID-positive count | Continuous (range: 1-7) |
| EQ-5D index (0.002-0.953) | Continuous (range: 0.002-0.953)  Scale: Health-related quality of life was assessed with the EQ-5D-5L scale, which covers mobility, self-care, usual activities, pain/discomfort, and anxiety/depression. Each dimension is rated from no problems (1) to extreme problems (5), resulting in an index value ranging from −0.391 (worst) to 1.0 (full health) based on the Chinese 5L value set [25]. The Cronbach's α value for the scale was 0.81 [26], and it was 0.84 in our sample.The Cronbach's α value for the scale was 0.81.  Reference: Li D-L, Wang Z-T, Nie X-Y, et al. EQ-5D-5l population norms for China derived from a national health survey. Value in Health. 2024;27(8):1108-1120. |
| **Block 7: mental health and psychosocial factors** |  |
| SCS: cyberchondria (4–20) | Continuous (range: 4-20)  Scale: The Short Cyberchondria Scale (CSS) comprises five items, including general disturbance, excessiveness and reassurance seeking. Participants rate each item on a 5-point Likert scale (1 = never/rarely; 5 = most of the time). Total scores are calculated by summing all items, with higher scores indicating greater severity. In its original validation, Cronbach’s α = 0.77.  Reference: Jokić-Begić N, Mikac U, Čuržik D, Sangster Jokić C. The development and validation of the short cyberchondria scale (SCS). Journal of Psychopathology and Behavioral Assessment. 2019;41:662-676. |
| PHQ-9: depression (0–27) | Continuous (range:0-27)  Scale: Depression was measured using the 9-item Patient Health Questionnaire (PHQ-9), a 9-item self-administered questionnaire. Each item is scored from 0 (not at all) to 3 (nearly every day), with total scores ranging from 0 to 27. Higher scores indicate more severe depressive symptoms. The Chinese PHQ-9 demonstrated excellent internal consistency (Cronbach α=0.86), whereas our sample showed a Cronbach α of 0.92.  Reference: Wang W, Bian Q, Zhao Y, et al. Reliability and validity of the Chinese version of the Patient Health Questionnaire (PHQ-9) in the general population. General hospital psychiatry. 2014;36(5):539-544. |
| GAD-3: anxiety (0–9) | Continuous (range: 0-9)  Scale: Anxiety levels were assessed with the 3-item General Anxiety Disorder scale, comprising 3 items that evaluate generalized anxiety symptoms over the past 2 weeks. Items are scored from 0 to 3, resulting in total scores between 0 and 9. Higher scores reflect greater anxiety severity. The 3‑item GAD demonstrated excellent reliability and validity, achieving an AUC of 0.988 against the full GAD‑7 cutoff.  Reference: Wang F, Wu Y, Wang S, Du Z, Wu Y. Development of an optimal short form of the GAD-7 scale with cross-cultural generalizability based on Riskslim. Gen Hosp Psychiatry. Mar-Apr 2024;87:33-40. |
| PSS-4: perceived stress (4–20) | Continuous (range:4-20) Scale: Perceived stress was evaluated using the 4-item Perceived Stress Scale, a 4-item instrument measuring stress perceptions over the past month. Each item is scored from 0 to 4, with total scores ranging from 0 to 16. Higher scores indicate higher stress levels. The Chinese 4-item Perceived Stress Scale demonstrated strong psychometric properties (Cronbach's α=0.85), whereas our sample showed a Cronbach α of 0.87.  Reference: She Z, Li D, Zhang W, Zhou N, Xi J, Ju K. Three versions of the perceived stress scale: psychometric evaluation in a nationally representative sample of Chinese adults during the COVID-19 pandemic. International journal of environmental research and public health. 2021;18(16):8312. |
| ASRS-6: adhd symptoms (0–24) | Continuous (range: 0-24)  Scale: The ASRS-6 is a widely used brief screener for ADHD. Six items related to ADHD are rated on a 5-point Likert-type scale.  Reference: Kessler RC, Adler LA, Gruber MJ, Sarawate CA, Spencer T, Van Brunt DL. Validity of the World Health Organization Adult ADHD Self‐Report Scale (ASRS) Screener in a representative sample of health plan members. International journal of methods in psychiatric research. 2007;16(2):52-65. |
| CCBI-7: work burnout (7–35) | Continuous (range:7-35)  Scale: The Chinese CBI‑7 (C‑CBI‑7) is a 7‑item scale measuring adult work-related burnout across two dimensions—exhaustion and frustration—using statements on physical/emotional fatigue and negative work attitudes. Items are rated 1 (rarely/strongly disagree) to 5 (often/strongly agree), and we use total score to reflect overall burnout severity.  Reference: Fan S, Li Y, Dong F, et al. Study protocol: A national cross-sectional study on psychology and behaviour investigation of Chinese residents in 2024, PBICR. medRxiv. 2025:2025.01. 14.25320520. |
| RSS: rest intolerance (8–40) | Contious (range: 8-40)  Scale: The Rest Shame Scale (RSS) is a self-developed, 8-item measure arranged as a single-choice matrix. It assesses shame related to taking breaks across four dimensions: negative affect, social comparison, obsessive thinking, and cognitive bias. Each item is rated on a 5-point Likert scale (1 = strongly disagree to 5 = strongly agree), with higher scores indicating greater rest-related shame.  Reference: Fan S, Li Y, Dong F, et al. Study protocol: A national cross-sectional study on psychology and behaviour investigation of Chinese residents in 2024, PBICR. medRxiv. 2025:2025.01. 14.25320520. |
| MDS5: maladaptive daydreaming (0–100) | Continuous (range: 0-100)  Scale: The Maladaptive Daydreaming Short Form (MD-SF5) is a concise 5-item self-report screener for identifying clinically significant maladaptive daydreaming. Their responses are scored with an 11-point scale, ranging from 0% to 100%. The total score is calculated as the mean, with higher scores reflecting more severe maladaptive daydreaming. The MD-SF5 showed good internal consistency (Cronbach’s α = .89).  Reference: Soffer-Dudek N, Oh H. Maladaptive daydreaming: A shortened assessment measure and its mental health correlates in a large United States sample. Comprehensive psychiatry. 2024;129:152441. |
| BSMAS: social media addiction (6–30) | Continuous (range: 6-30) Scale: Bergen Social Media Addiction Scale (BSMAS) was administered to assess the level of one's social media addiction. Participants were asked to report their experiences in the use of social media within a 12-month period. The BSMAS consisted of 6 items (Griffiths, 2005). A 5-point Likert scale was adopted for rating (from 1 = “very rarely” to 5 = “very often”), a higher sum score obtained from the BSMAS indicated a higher likelihood of being addicted to social media. In the present study, the Cronbach's alpha coefficient for the scale in the overall sample was 0.82.  Reference: Yue H, Zhang X, Cheng X, Liu B, Bao H. Measurement invariance of the Bergen social media addiction scale across genders. Frontiers in Psychology. 2022;13:879259. |
| Personal existence(1–7) | Continuous (1–7 levels), where 1 = completely without meaning and purpose; 4 = neutral; and 7 = very meaningful and purposeful. |

**Notes:** Block 1, demographics and socioeconomics; Block 2, life adversity and stress; Block 3, personality; Block 4, health literacy and empowerment; Block 5, lifestyle behaviors; Block 6, physical health and exposures; Block 7, mental health and psychosocial factors.

**Table S2.** Unweighted and weighted sample sizes and weighted acceptance of immersive health-care technologies by province (n=35,861).

| Province | Unweighted n | Weighted n | Weighted acceptance (95% CI) |
| --- | --- | --- | --- |
| Jilin | 122 | 65 | 72.34 (66.28, 78.39) |
| Liaoning | 430 | 335 | 71.08 (67.80, 74.36) |
| Tianjin | 395 | 406 | 67.68 (64.23, 71.13) |
| Shanghai | 886 | 1225 | 67.38 (65.43, 69.33) |
| Jiangsu | 973 | 906 | 67.02 (64.72, 69.32) |
| Guangdong | 938 | 1168 | 66.15 (63.95, 68.35) |
| Hebei | 1040 | 888 | 64.75 (62.69, 66.81) |
| Anhui | 4641 | 6306 | 64.57 (63.72, 65.42) |
| Shandong | 4677 | 4689 | 64.50 (63.52, 65.48) |
| Henan | 1906 | 2094 | 64.41 (62.94, 65.89) |
| Hainan | 278 | 246 | 64.19 (60.61, 67.76) |
| Heilongjiang | 1521 | 1022 | 63.55 (61.76, 65.33) |
| Chongqing | 655 | 729 | 63.52 (61.27, 65.78) |
| Xinjiang | 226 | 231 | 63.29 (58.91, 67.67) |
| Zhejiang | 335 | 378 | 63.11 (59.65, 66.56) |
| Beijing | 941 | 1138 | 63.01 (60.92, 65.10) |
| Guangxi | 416 | 358 | 62.87 (59.43, 66.31) |
| Fujian | 1068 | 1341 | 62.27 (60.40, 64.15) |
| Sichuan | 2509 | 2958 | 61.98 (60.77, 63.19) |
| Ningxia | 794 | 939 | 61.49 (59.30, 63.68) |
| Inner Mongolia | 54 | 42 | 61.42 (50.71, 72.13) |
| Yunnan | 1154 | 762 | 61.00 (58.44, 63.56) |
| Hunan | 912 | 760 | 60.79 (58.38, 63.20) |
| Shaanxi | 326 | 172 | 60.64 (56.16, 65.12) |
| Qinghai | 393 | 405 | 59.54 (56.04, 63.05) |
| Hubei | 1017 | 877 | 59.44 (57.25, 61.63) |
| Gansu | 441 | 439 | 59.43 (56.23, 62.64) |
| Jiangxi | 3881 | 2881 | 59.26 (57.95, 60.57) |
| Tibet | 1317 | 440 | 57.38 (54.92, 59.83) |
| Shanxi | 607 | 725 | 56.52 (54.03, 59.01) |
| Guizhou | 563 | 451 | 55.96 (53.07, 58.85) |
| Macau | 12 | 22 | 55.18 (39.04, 71.31) |
| Hong Kong | 12 | 12 | 47.88 (28.95, 66.81) |

***Notes*:** CI, confidence interval.

**Table S3.** Unweighted and weighted counts and weighted mean acceptance of immersive health-care technologies by age group and gender (n=35,861).

| **Age group (years)** | **Female** | | | **Male** | | |
| --- | --- | --- | --- | --- | --- | --- |
|  | **n** | **Weighted n** | **Weighted acceptance (95% *CI*)** | **n** | **Weighted n** | **Weighted acceptance**  **(95% *CI*)** |
| 18-24 | 9302 | 1166 | 68.51 (68.05, 68.96) | 6103 | 1340 | 66.81 (66.22, 67.39) |
| 25-29 | 1832 | 1444 | 68.43 (67.34, 69.52) | 1393 | 1606 | 65.16 (63.87, 66.45) |
| 30-34 | 1263 | 1991 | 66.44 (65.09, 67.79) | 958 | 2120 | 67.37 (65.80, 68.93) |
| 35-39 | 1368 | 1588 | 64.48 (63.17, 65.80) | 991 | 1686 | 63.12 (61.51, 64.73) |
| 40-44 | 1325 | 1497 | 63.61 (62.33, 64.89) | 1037 | 1575 | 63.94 (62.42, 65.46) |
| 45-49 | 1595 | 1851 | 63.49 (62.23, 64.76) | 1409 | 1923 | 64.02 (62.71, 65.32) |
| 50-54 | 1105 | 1984 | 61.69 (60.16, 63.22) | 1070 | 2019 | 62.22 (60.74, 63.70) |
| 55-59 | 770 | 1671 | 60.31 (58.48, 62.15) | 751 | 1679 | 62.17 (60.33, 64.01) |
| 60-64 | 540 | 1206 | 60.64 (58.46, 62.82) | 554 | 1218 | 60.82 (58.73, 62.90) |
| 65-69 | 446 | 1244 | 60.20 (57.71, 62.68) | 453 | 1200 | 62.83 (60.41, 65.24) |
| 70-74 | 469 | 840 | 56.76 (54.23, 59.29) | 456 | 798 | 59.49 (57.05, 61.93) |
| 75-79 | 198 | 545 | 53.01 (49.10, 56.91) | 222 | 487 | 55.24 (51.70, 58.78) |
| 80-84 | 95 | 371 | 57.05 (51.92, 62.19) | 75 | 302 | 56.72 (50.39, 63.05) |
| 85-89 | 24 | 211 | 57.63 (46.24, 69.01) | 23 | 146 | 55.09 (43.12, 67.06) |
| ≥90 | 19 | 96 | 63.68 (53.43, 73.94) | 15 | 55 | 61.67 (50.11, 73.23) |

***Notes:*** CI: confidence interval.

**Table S4.** Unweighted and weighted sample sizes and weighted mean acceptance by health condition (n=35,861).

| **Condition** | **n** | **Weighted n** | **Weighted acceptance (95% *CI*)** |
| --- | --- | --- | --- |
| Overall | 35,861 | 35,861 | 63.11 (62.75–63.46) |
| Any condition | 16,729 | 19,397 | 61.15 (60.63–61.67) |
| Hypertension | 2816 | 5687 | 60.31 (59.23–61.39) |
| Diabetes | 993 | 1990 | 59.59 (57.70–61.48) |
| Hyperlipidemia | 791 | 1509 | 62.26 (60.19–64.33) |
| Coronary | 453 | 938 | 60.25 (57.61–62.89) |
| Stroke | 110 | 202 | 60.06 (54.25–65.88) |
| Respiratory | 511 | 770 | 59.48 (56.81–62.15) |
| Urinary | 253 | 403 | 58.31 (54.79–61.84) |
| Digestive | 814 | 1094 | 61.30 (59.12–63.49) |
| Osteoporosis | 798 | 1591 | 57.60 (55.45–59.75) |
| Arthritis | 1060 | 1944 | 58.15 (56.32–59.98) |
| Tumor | 196 | 292 | 61.87 (56.99–66.76) |
| Obesity | 6156 | 7585 | 61.90 (61.10–62.70) |
| Rare disease | 69 | 71 | 54.24 (45.40–63.07) |
| Anxiety | 6129 | 5160 | 59.32 (58.40–60.23) |
| Depression | 8192 | 6891 | 59.37 (58.58–60.16) |

***Notes:*** CI: confidence interval

**Table S5.** Hierarchical weighted linear regression of sociodemographic predictors (Block 1) on immersive health-care acceptance (n=35,861).

| **Block 1 variables** | **Standardized coefficient (95% *CI*)** | ***p*** | **Adjusted *p*** |
| --- | --- | --- | --- |
| Race: others vs. Han | -0.01 (-0.02, 0.00) | 0.158 | 0.225 |
| Religion: yes vs. no | -0.01 (-0.03, -0.00) | 0.012 | 0.032 |
| Occupation: employed vs. student | -0.04 (-0.07, -0.02) | 0.001 | 0.003 |
| Occupation: self-employed vs. student | -0.04 (-0.06, -0.02) | <.001 | <.001 |
| Occupation: unemployed vs. student | -0.01 (-0.02, 0.01) | 0.291 | 0.395 |
| Occupation: retired vs. student | -0.02 (-0.04, -0.00) | 0.023 | 0.054 |
| Education: secondary vs. primary or below | 0.02 (0.00, 0.04) | 0.018 | 0.045 |
| Education: undergraduate vs. primary or below | 0.05 (0.03, 0.07) | <.001 | <.001 |
| Education: graduate vs. primary or below | 0.05 (0.03, 0.06) | <.001 | <.001 |
| City tier: new first tier vs. others | 0.01 (0.00, 0.03) | 0.010 | 0.030 |
| City tier: first tier vs. others | 0.00 (-0.01, 0.01) | 0.776 | 0.819 |
| Residence duration | 0.02 (0.00, 0.03) | 0.010 | 0.030 |
| Age | -0.08 (-0.10, -0.06) | <.001 | <.001 |
| Handedness: right hand vs. left hand | 0.01 (0.00, 0.02) | 0.028 | 0.064 |
| Residence (last 3 months): urban vs. rural | 0.03 (0.02, 0.04) | <.001 | <.001 |
| Household registration: urban vs. rural | 0.01 (-0.00, 0.03) | 0.059 | 0.113 |
| Relationship status: in relationship vs. single | -0.01 (-0.03, 0.01) | 0.307 | 0.406 |
| Relationship status: married vs. single | -0.01 (-0.03, 0.02) | 0.588 | 0.684 |
| Relationship status: divorced vs. single | 0.01 (-0.00, 0.02) | 0.052 | 0.103 |
| Relationship status: widowed vs. single | -0.01 (-0.02, 0.00) | 0.107 | 0.170 |
| Family type: single-parent family vs. single-person family | -0.02 (-0.04, 0.00) | 0.081 | 0.134 |
| Family type: couple only vs. single-person family | -0.08 (-0.11, -0.04) | <.001 | <.001 |
| Family type: DINK vs. single-person family | -0.00 (-0.02, 0.01) | 0.742 | 0.798 |
| Family type: nuclear family vs. single-person family | -0.03 (-0.07, 0.01) | 0.191 | 0.265 |
| Family type: extended family vs. single-person family | -0.04 (-0.07, -0.00) | 0.044 | 0.090 |
| Family type: joint family vs. single-person family | -0.01 (-0.03, 0.00) | 0.120 | 0.180 |
| Family type: skipped-generation family vs. single-person family | -0.04 (-0.05, -0.02) | <.001 | <.001 |
| Family type: other vs. single-person family | -0.02 (-0.04, -0.00) | 0.041 | 0.087 |
| Birth sex: male vs. female | -0.03 (-0.07, 0.01) | 0.115 | 0.178 |
| Birth sex: others vs. female | -0.02 (-0.03, -0.01) | 0.005 | 0.019 |
| Sex orientation: bisexual vs. heterosexual | -0.00 (-0.02, 0.01) | 0.682 | 0.756 |
| Sex orientation: homosexual vs. heterosexual | 0.01 (-0.00, 0.03) | 0.082 | 0.134 |
| Sex orientation: other vs. heterosexual | -0.02 (-0.03, -0.00) | 0.009 | 0.030 |
| Sex orientation: asexual vs. heterosexual | -0.00 (-0.02, 0.01) | 0.436 | 0.552 |
| Gender identity: genderqueer/genderfluid vs. female | -0.00 (-0.02, 0.01) | 0.812 | 0.826 |
| Gender identity: male vs. female | 0.03 (-0.00, 0.07) | 0.078 | 0.134 |
| Gender identity: others vs. female | -0.01 (-0.02, 0.01) | 0.399 | 0.517 |
| Gender identity: transgender female (male-to-female) vs. female | -0.00 (-0.02, 0.02) | 0.812 | 0.826 |
| Gender identity: transgender male (female-to-male) vs. female | -0.00 (-0.02, 0.01) | 0.536 | 0.650 |
| Lived alone in past 3 months: yes vs. no | -0.00 (-0.02, 0.01) | 0.616 | 0.703 |
| Number of siblings | -0.07 (-0.08, -0.05) | <.001 | <.001 |
| House area | 0.04 (0.03, 0.06) | <.001 | <.001 |
| House structure: number of bedrooms | 0.00 (-0.01, 0.02) | 0.971 | 0.971 |
| Number of properties owned | 0.01 (-0.00, 0.03) | 0.073 | 0.129 |
| No debt: yes vs. no | 0.02 (0.00, 0.03) | 0.016 | 0.042 |
| Per-person income | 0.01 (0.00, 0.03) | 0.034 | 0.075 |
| Medical insurance: only rural vs. no medicare | 0.01 (-0.01, 0.03) | 0.485 | 0.601 |
| Medical insurance: only resident vs. no medicare | 0.05 (0.02, 0.08) | 0.003 | 0.011 |
| Medical insurance: only employee vs. no medicare | 0.03 (0.01, 0.06) | 0.010 | 0.030 |
| Medical insurance: only medicare public vs. no medicare | 0.02 (-0.00, 0.04) | 0.071 | 0.129 |
| Medical insurance: only commercial vs. no medicare | 0.00 (-0.01, 0.02) | 0.570 | 0.677 |
| Medical insurance: two medicare vs. no medicare | 0.07 (0.04, 0.09) | <.001 | <.001 |
| Medical insurance: more than two medicare vs. no medicare | 0.04 (0.02, 0.05) | <.001 | <.001 |
| Medical cost difficulty: yes vs. no | -0.03 (-0.04, -0.02) | <.001 | <.001 |
| Medicare enrollment location: living area vs. Hukou location | 0.01 (-0.00, 0.02) | 0.143 | 0.208 |
| Medicare enrollment location: others vs. Hukou location | -0.00 (-0.02, 0.01) | 0.690 | 0.756 |
| Social status | 0.17 (0.16, 0.18) | <.001 | <.001 |

**Table S6.** Hierarchical weighted linear regression of life adversity and adverse childhood experience predictors (Block 2) on immersive health-care acceptance (n=35,861).

| **Block 2 variables** | **Standardized coefficient (95% *CI*)** | ***p*** | ***Adjusted p*** |
| --- | --- | --- | --- |
| Life event: immediate family death: yes vs. no | -0.01 (-0.02, 0.00) | 0.081 | 0.134 |
| Life event: unemployment: yes vs. no | 0.00 (-0.01, 0.01) | 0.649 | 0.729 |
| Life event: legal dispute involvement: yes vs. no | 0.01 (0.00, 0.03) | 0.009 | 0.022 |
| Life event: high study/work stress: yes vs. no | 0.04 (0.02, 0.06) | <.001 | <.001 |
| Life event: family economic hardship: yes vs. no | 0.00 (-0.01, 0.02) | 0.686 | 0.747 |
| Life event: unable to reunite with family: yes vs. no | -0.00 (-0.02, 0.01) | 0.569 | 0.659 |
| Life event: major change in routine: yes vs. no | 0.00 (-0.01, 0.01) | 0.935 | 0.962 |
| Life event: family conflict: yes vs. no | 0.01 (-0.00, 0.02) | 0.165 | 0.240 |
| Life event: difficulty purchasing supplies: yes vs. no | -0.02 (-0.04, -0.01) | <.001 | 0.001 |
| Life event: accident or natural disaster: yes vs. no | -0.01 (-0.02, 0.00) | 0.309 | 0.396 |
| Life event: theft or property loss: yes vs. no | 0.00 (-0.01, 0.01) | 0.785 | 0.831 |
| Life event: serious injury or illness: yes vs. no | 0.03 (0.02, 0.04) | <.001 | <.001 |
| Life event: other: yes vs. no | -0.02 (-0.03, -0.00) | 0.007 | 0.018 |
| Life event: none: yes vs. no | -0.01 (-0.03, 0.01) | 0.521 | 0.614 |
| ACE: psychological abuse (0–2) | 0.07 (0.05, 0.09) | <.001 | <.001 |
| ACE: emotional neglect: yes vs. no | 0.04 (0.03, 0.06) | <.001 | <.001 |
| ACE: physical abuse (0–2) | -0.05 (-0.06, -0.03) | <.001 | <.001 |
| ACE: sexual abuse (0–4) | -0.08 (-0.10, -0.06) | <.001 | <.001 |
| ACE: father died before age 18: yes vs. no | -0.01 (-0.03, 0.01) | 0.257 | 0.355 |
| ACE: mother died before age 18: yes vs. no | -0.01 (-0.02, 0.00) | 0.274 | 0.371 |
| ACE: older brother died before age 18: yes vs. no | -0.01 (-0.02, -0.00) | 0.007 | 0.018 |
| ACE: younger brother died before age 18: yes vs. no | -0.00 (-0.01, 0.01) | 0.665 | 0.735 |
| ACE: older sister died before age 18: yes vs. no | 0.00 (-0.01, 0.01) | 0.501 | 0.599 |
| ACE: younger sister died before age 18: yes vs. no | -0.01 (-0.02, 0.00) | 0.139 | 0.218 |
| ACE: no relative died before age 18: yes vs. no | 0.02 (-0.00, 0.04) | 0.066 | 0.118 |
| ACE: witnessed maternal violence (0–4) | -0.01 (-0.03, 0.01) | 0.283 | 0.375 |
| ACE: community violence exposure (0–2) | 0.02 (0.00, 0.03) | 0.031 | 0.058 |
| ACE: collective violence exposure (0–2) | -0.05 (-0.07, -0.03) | <.001 | <.001 |
| ACE: economic environment (4–20) | 0.06 (0.04, 0.07) | <.001 | <.001 |
| Socioeconomic status (youth) (1–7) | 0.14 (0.13, 0.16) | <.001 | <.001 |

***Notes*:** ACE, adverse childhood experiences.

**Table S7.** Hierarchical weighted linear regression of personality predictors (Block 3) on immersive health-care acceptance (n=35,861).

| **Block 3 variables** | **Standardized coefficient (95% *CI*)** | ***p*** | ***Adjusted p*** |
| --- | --- | --- | --- |
| Personality (extraversion) (2–10) | -0.00 (-0.01, 0.01) | 0.587 | 0.649 |
| Personality (agreeableness) (2–10) | 0.00 (-0.01, 0.01) | 0.748 | 0.799 |
| Personality (conscientiousness) (2–10) | 0.03 (0.02, 0.04) | <.001 | <.001 |
| Personality (neuroticism) (2–10) | -0.00 (-0.01, 0.01) | 0.566 | 0.649 |
| Personality (openness) (2–10) | -0.02 (-0.03, -0.01) | <.001 | 0.001 |
| NGSES: self-efficacy (3–15) | 0.10 (0.09, 0.12) | <.001 | <.001 |
| NARQ: narcissistic admiration & rivalry (6–36) | 0.04 (0.03, 0.05) | <.001 | <.001 |

***Notes*:** CI, confidence interval; NGSES, New General Self-Efficacy Scale: NARQ, Narcissistic Admiration and Rivalry Questionnaire.

**Table S8.** Hierarchical weighted linear regression of literacy and empowerment predictors (Block 4) on immersive health-care acceptance (n=35,861).

| **Block 4 variables** | **Standardized coefficient (95% *CI*)** | ***p*** | ***Adjusted p*** |
| --- | --- | --- | --- |
| Family-neighbor relationship (1–7) | 0.05 (0.04, 0.07) | <.001 | <.001 |
| Have someone to trust: yes vs. no | -0.01 (-0.02, 0.00) | 0.177 | 0.260 |
| eHEALS: ehealth literacy (5–25) | 0.09 (0.07, 0.10) | <.001 | <.001 |
| FHS-SF: family health (10–50) | 0.01 (-0.01, 0.03) | 0.218 | 0.309 |
| HLS-SF: health literacy (0–12) | 0.01 (0.00, 0.03) | 0.044 | 0.079 |
| Received help when needed: yes vs. no | 0.00 (-0.01, 0.02) | 0.384 | 0.491 |
| Social isolation (3–18) | 0.01 (-0.00, 0.03) | 0.080 | 0.130 |
| Social loneliness (2–12) | -0.02 (-0.03, -0.01) | 0.005 | 0.011 |
| Social connection (5–30) | -0.01 (-0.02, 0.01) | 0.255 | 0.355 |
| PSSS: perceived social support (3–21) | 0.04 (0.02, 0.06) | <.001 | <.001 |
| Implicit health beliefs (6–36) | 0.01 (-0.00, 0.02) | 0.181 | 0.261 |
| FCS-SF: family communication (4–20) | 0.03 (0.01, 0.04) | <.001 | <.001 |
| SREBQ: dietary self-regulation (5–25) | 0.04 (0.03, 0.05) | <.001 | <.001 |
| Antibiotic knowledge: yes vs. no | 0.00 (-0.01, 0.01) | 0.760 | 0.801 |
| Past digital health intervention use: yes vs. no | 0.15 (0.14, 0.16) | <.001 | <.001 |

***Notes*:** eHEALS, eHealth Literacy Scale; FHS‑SF, Family Health Scale‑Short Form; HLS‑SF, Health Literacy Survey‑Short Form; PSSS, Perceived Social Support Scale; FCS‑SF, Family Communication Scale‑Short Form; SREBQ, Self‑Regulation of Eating Behavior Questionnaire.

**Table S9.** Hierarchical weighted linear regression of lifestyle factor predictors (Block 5) on immersive health-care acceptance (n=35,861).

| **Block 5 variables** | **Standardized coefficient (95% *CI*)** | ***p*** | ***Adjusted p*** |
| --- | --- | --- | --- |
| Smoking habit: former smoker vs. no | -0.01 (-0.02, 0.00) | 0.215 | 0.307 |
| Smoking habit: yes (conventional cigarettes) vs. no | -0.03 (-0.04, -0.02) | <.001 | <.001 |
| Smoking habit: yes (e-cigarettes) vs. no | -0.02 (-0.03, -0.00) | 0.008 | 0.020 |
| Smoking habit: yes (both) vs. no | -0.02 (-0.03, -0.00) | 0.010 | 0.020 |
| Alcohol use: new drinker vs. never drink | -0.01 (-0.02, 0.01) | 0.319 | 0.402 |
| Alcohol use: current drinker (including occasional) vs. never drink | 0.00 (-0.01, 0.01) | 0.904 | 0.914 |
| Alcohol use: former drinker vs. never drink | 0.00 (-0.01, 0.01) | 0.893 | 0.912 |
| MET category: moderate vs. low | -0.02 (-0.03, -0.01) | 0.004 | 0.011 |
| MET category: high vs. low | 0.00 (-0.01, 0.02) | 0.602 | 0.657 |
| Chronotype: morning vs. evening | -0.03 (-0.04, -0.01) | <.001 | 0.001 |
| Sleep hours: 6–7 hours vs. ≤6 hours | 0.03 (0.02, 0.05) | <.001 | <.001 |
| Sleep hours: 7–8 hours vs. ≤6 hours | 0.03 (0.01, 0.05) | 0.001 | 0.002 |
| Sleep hours: 8–9 hours vs. ≤6 hours | 0.00 (-0.01, 0.02) | 0.968 | 0.968 |
| Sleep hours: >9 hours vs. ≤6 hours | 0.01 (-0.00, 0.03) | 0.105 | 0.161 |
| Sleep difficulty: sometimes vs. never/rarely | -0.03 (-0.04, -0.01) | <.001 | <.001 |
| Sleep difficulty: often vs. never/rarely | 0.00 (-0.01, 0.01) | 0.635 | 0.685 |
| Snore: yes vs. no | 0.01 (-0.00, 0.02) | 0.255 | 0.334 |
| Daytime sleepiness: sometimes vs. never/rarely | -0.03 (-0.04, -0.02) | <.001 | <.001 |
| Daytime sleepiness: often vs. never/rarely | -0.02 (-0.03, -0.01) | 0.004 | 0.010 |
| Daytime sleepiness: always vs. never/rarely | -0.01 (-0.02, 0.00) | 0.142 | 0.206 |
| Stable sleep duration: somewhat unstable vs. very unstable | 0.03 (0.00, 0.05) | 0.037 | 0.067 |
| Stable sleep duration: somewhat stable vs. very unstable | 0.07 (0.03, 0.10) | <.001 | 0.001 |
| Stable sleep duration: very stable vs. very unstable | 0.02 (-0.01, 0.06) | 0.230 | 0.319 |
| Extra salt in food: sometimes vs. never/rarely | -0.02 (-0.03, -0.01) | 0.001 | 0.003 |
| Extra salt in food: often vs. never/rarely | -0.03 (-0.04, -0.02) | <.001 | <.001 |
| Extra salt in food: always vs. never/rarely | -0.03 (-0.04, -0.01) | <.001 | <.001 |
| Exposure to music: yes vs. no | -0.01 (-0.02, 0.01) | 0.255 | 0.334 |
| Exposure to art: yes vs. no | 0.04 (0.02, 0.05) | <.001 | <.001 |
| Exposure to dance: yes vs. no | 0.01 (-0.01, 0.02) | 0.414 | 0.502 |
| Exposure to other arts: yes vs. no | 0.01 (-0.00, 0.02) | 0.102 | 0.160 |

***Notes:*** CI, confidence interval; MET, metabolic equivalent of task.

**Table S10.** Hierarchical weighted linear regression of physical health predictors (Block 6) on immersive health-care acceptance (n=35,861).

| **Block 6 variables** | **Standardized coefficient (95% *CI*)** | ***p*** | ***Adjusted p*** |
| --- | --- | --- | --- |
| BMI classification: underweight vs. severely underweight | 0.01 (-0.02, 0.04) | 0.580 | 0.673 |
| BMI classification: normal weight vs. severely underweight | 0.00 (-0.05, 0.05) | 0.941 | 0.948 |
| BMI classification: overweight (Asian) vs. severely underweight | 0.01 (-0.03, 0.05) | 0.488 | 0.598 |
| BMI classification: obesity (Asian) vs. severely underweight | 0.00 (-0.03, 0.04) | 0.852 | 0.910 |
| Hypertension diagnosis: yes vs. no | 0.00 (-0.01, 0.01) | 0.813 | 0.896 |
| Diabetes diagnosis: yes vs. no | -0.00 (-0.01, 0.00) | 0.227 | 0.327 |
| Hyperlipidemia diagnosis: yes vs. no | 0.02 (0.01, 0.03) | <.001 | <.001 |
| Coronary heart disease diagnosis: yes vs. no | 0.00 (-0.00, 0.01) | 0.503 | 0.605 |
| Stroke diagnosis: yes vs. no | 0.01 (-0.00, 0.01) | 0.118 | 0.195 |
| Respiratory disease diagnosis: yes vs. no | -0.01 (-0.02, 0.00) | 0.133 | 0.206 |
| Urinary disease diagnosis: yes vs. no | -0.01 (-0.01, 0.00) | 0.224 | 0.327 |
| Digestive disease diagnosis: yes vs. no | -0.00 (-0.01, 0.00) | 0.353 | 0.454 |
| Osteoporosis diagnosis: yes vs. no | -0.01 (-0.02, 0.00) | 0.129 | 0.206 |
| Arthritis diagnosis: yes vs. no | -0.02 (-0.03, -0.01) | 0.001 | 0.003 |
| Tumor diagnosis: yes vs. no | 0.00 (-0.01, 0.01) | 0.845 | 0.910 |
| Rare disease diagnosis: yes vs. no | -0.01 (-0.02, 0.00) | 0.091 | 0.166 |
| Other diagnosis: yes vs. no | -0.01 (-0.02, 0.01) | 0.337 | 0.439 |
| No diagnosis: yes vs. no | -0.01 (-0.03, 0.00) | 0.109 | 0.182 |
| Injury event: motor vehicle accident: yes vs. no | -0.01 (-0.02, 0.00) | 0.103 | 0.174 |
| Injury event: non‐motor vehicle accident: yes vs. no | 0.00 (-0.01, 0.02) | 0.533 | 0.630 |
| Injury event: fall or falling: yes vs. no | 0.00 (-0.01, 0.02) | 0.891 | 0.927 |
| Injury event: blunt instrument injury: yes vs. no | -0.01 (-0.02, -0.00) | 0.050 | 0.101 |
| Injury event: firearm injury: yes vs. no | 0.02 (0.01, 0.03) | 0.001 | 0.003 |
| Injury event: sharp‐object injury: yes vs. no | 0.01 (0.00, 0.03) | 0.038 | 0.077 |
| Injury event: burn or scald: yes vs. no | 0.02 (0.01, 0.04) | 0.001 | 0.004 |
| Injury event: suffocation or hanging: yes vs. no | -0.01 (-0.02, 0.00) | 0.195 | 0.287 |
| Injury event: drowning: yes vs. no | -0.01 (-0.02, 0.00) | 0.070 | 0.134 |
| Injury event: poisoning: yes vs. no | -0.00 (-0.01, 0.01) | 0.932 | 0.947 |
| Injury event: animal‐related injury: yes vs. no | -0.00 (-0.01, 0.01) | 0.916 | 0.945 |
| Injury event: sexual assault: yes vs. no | 0.01 (-0.00, 0.03) | 0.072 | 0.135 |
| Injury event: other: yes vs. no | 0.01 (-0.00, 0.02) | 0.128 | 0.206 |
| Injury event: none: yes vs. no | 0.00 (-0.02, 0.03) | 0.854 | 0.910 |
| HPV vaccination: yes vs. no | 0.01 (0.00, 0.03) | 0.037 | 0.077 |
| Flu vaccination: yes vs. no | -0.02 (-0.03, -0.00) | 0.011 | 0.029 |
| Shingles vaccination: yes vs. no | 0.01 (-0.01, 0.02) | 0.366 | 0.466 |
| Hepatitis vaccination: yes vs. no | 0.02 (0.01, 0.03) | 0.001 | 0.004 |
| COVID vaccination: yes vs. no | 0.03 (0.00, 0.05) | 0.019 | 0.047 |
| No vaccination: yes vs. no | 0.01 (-0.01, 0.03) | 0.479 | 0.593 |
| COVID-positive count | -0.02 (-0.03, -0.01) | <.001 | 0.001 |
| EQ-5D index (0.002–0.953) | 0.01 (-0.00, 0.03) | 0.063 | 0.121 |

***Notes***: BMI, body mass index; CI, confidence interval; HPV, human papillomavirus; COVID, coronavirus disease 2019; EQ‑5D, EuroQol five‑dimension health index.

**Table S11.** Hierarchical weighted linear regression of mental-health predictors (Block 7) on immersive health-care acceptance (n=35,861).

| **Block 7 variables** | **Standardized coefficient (95% *CI*)** | ***p*** | ***Adjusted p*** |
| --- | --- | --- | --- |
| SCS: cyberchondria (4–20) | 0.03 (0.01, 0.04) | <.001 | 0.001 |
| PHQ-9: depression (0–27) | -0.04 (-0.06, -0.02) | 0.001 | 0.002 |
| GAD-3: anxiety (0–9) | 0.00 (-0.02, 0.02) | 0.872 | 0.932 |
| PSS-4: perceived stress (4–20) | 0.02 (0.00, 0.04) | 0.031 | 0.053 |
| ASRS-6: ADHD symptoms (0–24) | -0.07 (-0.09, -0.05) | <.001 | <.001 |
| CCBI-7: work burnout (7–35) | 0.05 (0.04, 0.07) | <.001 | <.001 |
| RSS: rest intolerance (8–40) | 0.01 (-0.00, 0.03) | 0.083 | 0.120 |
| MDS5: maladaptive daydreaming (0–100) | -0.02 (-0.03, -0.00) | 0.033 | 0.055 |
| BSMAS: social media addiction (6–30) | 0.05 (0.04, 0.06) | <.001 | <.001 |
| Personal existence (1–7) | 0.06 (0.04, 0.07) | <.001 | <.001 |

***Notes***: SCS, Short Cyberchondria Scale; PHQ-9, Patient Health Questionnaire-9; GAD-7, Generalized Anxiety Disorder-7; PSS-4, Perceived Stress Scale-4; ADHD, attention-deficit/hyperactivity disorder; ASRS-6, 6-item Adult ADHD Self-Report Scale; CCBI-7, 7-item Chinese version of Copenhagen Burnout Inventory; RSS, Rest Shame Scale; MDS-5, 5-item Maladaptive Daydreaming Short Form; BSMAS, Bergen Social Media Addiction Scale.
